# Characterization of microRNAs Identified in a Table Grapevine Cultivar with Validation of Computationally Predicted Grapevine miRNAs by miR-RACE

**DOI:** 10.1371/journal.pone.0021259

**Published:** 2011-07-28

**Authors:** Chen Wang, Lingfei Shangguan, Korir Nicholas Kibet, Xicheng Wang, Jian Han, Changnian Song, Jinggui Fang

**Affiliations:** College of Horticulture, Nanjing Agricultural University, Nanjing City, Jiangsu Province, China; Cardiovascular Research Institute Maastricht, Maastricht University, The Netherlands

## Abstract

**Background:**

Alignment analysis of the Vv-miRNAs identified from various grapevine cultivars indicates that over 30% orthologous Vv-miRNAs exhibit a 1–3 nucleotide discrepancy only at their ends, suggesting that this sequence discrepancy is not a random event, but might mainly derive from divergence of cultivars. With advantages of miR-RACE technology in determining precise sequences of potential miRNAs from bioinformatics prediction, the precise sequences of vv-miRNAs predicted computationally can be verified with miR-RACE in a different grapevine cultivar. This presents itself as a new approach for large scale discovery of precise miRNAs in different grapevine varieties.

**Methodology/Principal Findings:**

Among 88 unique sequences of Vv-miRNAs from bioinformatics prediction, 83 (96.3%) were successfully validated with MiR-RACE in grapevine cv. ‘Summer Black’. All the validated sequences were identical to their corresponding ones obtained from deep sequencing of the small RNA library of ‘Summer Black’. Quantitative RT-PCR analysis of the expressions levels of 10 Vv-miRNA/target gene pairs in grapevine tissues showed some negative correlation trends. Finally, comparison of Vv-miRNA sequences with their orthologs in *Arabidopsis* and study on the influence of divergent bases of the orthologous miRNAs on their targeting patterns in grapevine were also done.

**Conclusion:**

The validation of precise sequences of potential Vv-miRNAs from computational prediction in a different grapevine cultivar can be a new way to identify the orthologous Vv-miRNAs. Nucleotide discrepancy of orthologous Vv-miRNAs from different grapevine cultivars normally does not change their target genes. However, sequence variations of some orthologous miRNAs in grapevine and *Arabidopsis* can change their targeting patterns. These precise Vv-miRNAs sequences validated in our study could benefit some further study on grapevine functional genomics.

## Introduction

MicroRNAs (miRNAs) are a newly identified class of endogenous tiny, non-protein-coding RNA molecules that play very important roles in post transcriptional gene regulation through degradation of target mRNAs or by repression of targeted gene translation in organisms [1]–[5]. MiRNAs could be implicated in many cellular processes, such as growth, development, differentiation and proliferation as well as apoptosis by modulating the expression of their target endogenous genes [6]–[8]. In plants, miRNAs are transcribed by RNA polymerase II into long primary transcripts (pri-miRNAs) [9],[10], which are cut into miRNA precursors (pre-miRNAs) with typical hairpin structure(s). Mature miRNAs are generated from the stem portion of the single stranded stem-loop precursor by the complex containing the nuclear RNase III enzyme and the ribonuclease III-like enzyme Dicer (DCL1) [11], then the mature miRNA is incorporated into the RNA-induced silencing complex (RISC) and guides RISC to complementary mRNA targets. Finally, the RISC inhibits translation elongation or triggers the degradation of target mRNA [12]. Recently, hundreds of miRNAs from diverse plants have been discovered, among which many are highly evolutionarily conserved between species, ranging from mosses to higher flowering eudicot species in the plant kingdom [10], [13]. The high evolutionary conservation of miRNA sequences within the plant kingdom provides powerful evidence supporting prediction and validation of conserved miRNAs and their target genes from all plant species by bioinformatics and experimental methods [14], [15].

With the advance of bioinformatics, many computational methods have been employed for the prediction and identification of miRNAs [16]–[19]. A great number of plant miRNAs predicted computationally have been deposited in miRBase (http://www.mirbase.org/), such as *Arabidopsis thaliana* (190), *Populus trichocarpa* (234), *Vitis vinifera* (137), *Oryza sativa* (414) and *Zea mays* (109). Theoretically, computational prediction of miRNA can discover all the potential miRNAs based on the criteria set for characterization of miRNAs. However, it seems that some false predictions are unavoidable due to the evolution of miRNAs and the non-availability of miRNA end characteristics that exactly define the start and stop codes of mature miRNAs. This is also the main disadvantage of computational prediction compared to direct cloning of miRNAs. To the best of our knowledge, there are few studies on comprehensive determination of precise sequences of computationally predicted miRNAs. Earlier related studies were mainly focused on determining the expression of miRNAs using Northern blotting and/or RT-PCR techniques which despite being robust can only confirm the existence and size, but not the full precise sequence of a miRNA to be identified. At present, the newly reported miR-RACE can overcome the shortcoming mentioned above in that it is preferentially suitable in validation of precise sequences, especially both ends, of computationally predicted miRNAs in an organism, which is exemplified by successful utilization in earlier studies on identification of citrus and apple miRNA precise sequences [20], [21]. This validation of the precise sequences of computationally predicted miRNAs can lay a solid foundation for some downstream research applications, such as accurate and powerful prediction of miRNA targets, miRNA evolution, miRNA's function on gene expression, and the mechanism of miRNA biogenesis.

Grapevine cultivated for both fruit and beverage has long been playing an important role in human diet and health, and it is also among the most economically important fruit crops worldwide. It is now growing to be a preferable experimental system in fruit crops as it is the first fruit crop to get its genome fully sequenced [22], [23]. The released grapevine genomic sequence together with the abundance of grapevine expressed sequence tags (ESTs) can be of benefit in bioinformatics prediction of miRNAs and their target genes for the elucidation of gene expression, from which potential Vv-miRNAs have been identified [24]–[26]. In contrast, the total number of the Vv-miRNAs identified from sequencing of small RNA libraries and direct cloning [24]–[26] is still fewer than those predicted computationally. Interestingly, over 30% orthologous Vv-miRNAs from different grapevine variety populations (*Vitis vinifera* and hybrids of *V. vinifera* and *V. labrusca* (GSE24531) [24]–[26] show divergence of 1–3 bases only at either ends of their sequences. This phenomenon could suggest that it would be still essential to identify more Vv-miRNAs from different grapevine cultivars, and the validation of precise end sequences of the predicted Vv-miRNAs in different grapevine cultivars could be developed as a new strategy for identification of Vv-miRNAs. Such a novel strategy would be both efficient and powerful. Considering there was no earlier report on identification of miRNAs in table grapevines, we employed the miR-RACE technology for the first time to develop a new strategy for identification of miRNAs in grapevine cv. “Summer Black”, a cultivar cultivated mainly as a table grape, along with validation of precise sequences of 162 computationally identified Vv-miRNAs which is the largest group of Vv-miRNAs on record.

## Results

### Validation of Vv-miRNAs in a table grapevine cultivar using miR-RACE

In this study, a grapevine cv. ‘Summer Black’ and the Vv-miRNAs predicted computationally were our preferable choices to be researched on, since the former is one of the most popular and important grapevine cultivars grown for a table grape in China and the later is the largest group of candidate Vv-miRNAs released publicly. A total of 162 predicted Vv-miRNAs with 88 unique sequences belonging to 31 miRNA families were researched, among which 114 were predicted by Jallion *et al.* [22], 22 predicted by Cai *et al.* [27], and the other 26 were predicted by both of them. In the last group of 26 Vv-miRNAs, 14 were identical in both reports while another 12 exhibited some different nucleotides in the end sequences from the two studies. These 162 predicted Vv-miRNAs can be used as baits in the discovery of their corresponding orthologs in other grapevine cultivars (e.g “Summer Black” in our study) by validating the termini nucleotides based on the phenomenon that the nucleotide variations only happen at either ends of the corresponding Vv-miRNAs in different grapevine cultivars. From identification of the 162 Vv-miRNAs with miR-RACE technology in ‘Summer Black’, a total of 83 unique sequences (94.3%) comprising 156 Vv-miRNAs, could be successfully amplified, sequenced and spliced into their precise sequences as listed in Table S1, and the profiles of 3′ RACE and 5′ RACE products of eight Vv-miRNAs are shown as example (Fig. S1). The remaining 5 unique sequences of 6 Vv-miRNAs (Vv-miR172a/b, Vv-miR477-3p, Vv-mir845a/b, Vv-miR845c) could neither be identified by both miR-RACE and in our deep sequencing studies, probably due to their too low abundance or they might have been not-true-to-type in grapevine. Furthermore, among the 83 unique sequences validated in “Summer Black”, 60 (73.5%) unique sequences of 113 Vv-miRNAs could be confirmed to be same as those predicted computationally, whereas the other 23 unique Vv-miRNA sequences exhibited some discrepancies of 1–3 termini nucleotides (Table S1). Alignment analysis of the V-miRNAs validated here in “Summer Black” with those from our Solexa sequencing of small RNA library from the same grapevine variety revealed that all 130 corresponding conserved Vv-miRNAs (GEO No. GSE24531) from Solexa sequencing and sequence validation by miR-RACE were identical, suggesting the accuracy and workability of miR-RACE in the discovery of miRNAs in grapevine “Summer Black”. All the 83 unique sequences of 156 Vv-miRNAs validated here can be considered as the true-to-type in grapevine “Summer Black”. The importance of sequence validation of Vv-miRNAs was also indicated from the validation of the 12 ones with some divergence in sequences predicted both by Jallion *et al.* [22] and Cai *et al.* [27], among which two Vv-miRNAs (Vv-miR169w and Vv-miR169v) predicted by Jallion *et al.* [22] and 4 (Vv-miR169q, Vv-miR169o, Vv-miR477a and Vv-miR482) predicted by Cai *et al.* [27] were confirmed to be the true-to-type. Another 6 (Vv-miR169b, Vv-miR169h, Vv-miR393b, Vv-miR393i, Vv-miR845a and Vv-miR845b) were validated to be those predicted wrongly in both studies (Table S1).

### Alignment analysis of orthologous Vv-miRNA sequences

In order to characterize the orthologous Vv-miRNAs in various grapevine cultivars, 121 Vv-miRNAs verified in this work were further compared with their orthologs deposited in miRbase 15.0 (April, 2010), among which the later group belonging to 56 unique sequences were those identified in grapevine cultivar ‘Pinot Noir’ clone PN40024 [25]. Thirty unique orthologous sequences were aligned and found to be identical, but the other 26 exhibited some divergence of their termini nucleotides (Table 1). Similarly, comparison with 11 unique orthologous miRNA sequences verified in grapevine variety ‘Nebbiolo’ [26], nine were identical to those sequenced by miR-RACE, while the other 2 miRNA (Vv-miR166b and Vv-miR167a) sequences showed 1 and 2 base differences at either or both ends from the orthologous sequences, respectively. All these findings powerfully demonstrate the fact that there indeed exist some differences in sequences of orthologous Vv-miRNAs between different varieties of grapevine, and this might cause the discrepancy of functions of some Vv-miRNAs during grapevine speciation, which calls for additional studies to be carried out.

**Table 1:** Sequences of miRNAs validated experimentally from table and wine grapevine cultivars.

MiRNA	Sequences identified in ‘Summer Black’	Sequences identified in ‘Pinot Noir’ clone PN40024 (miRbase 15.0)
Vv-miR156i	UGACAGAAGAUAGAGAGCAC	***U***UGACAGAAGAUAGAGAGCAC
Vv-miR159a	**∧** UUGGAGUGAAGGGAGCUCUC	***C***UUGGAGUGAAGGGAGCUCUC
Vv-miR160a,b	UGCCUGGCUCCCUGAAUGCCA	UGCCUGGCUCCCUGAAUGCCA***UC***
Vv-miR166a	UCGGACCAGGCUUCAUUCC***UG***	UCGGACCAGGCUUCAUUCC
Vv-miR166b	UCGGACCAGGCUUCAUUCC***UC***	UCGGACCAGGCUUCAUUCC
Vv-miR166c,e,h	UCGGACCAGGCUUCAUUCCCC***C***	UCGGACCAGGCUUCAUUCCCC
Vv-miR166d,f,g	UCGGACCAGGCUUCAUUCCCC***U***	UCGGACCAGGCUUCAUUCCCC
Vv-miR167c	UGCCAAAGGAGAGUUGCCCU **∧**	UGAAGCUGCCAGCAUGAUCU***C***
Vv-miR169e	UAGCCAAGGAUGACUUGCCUG	UAGCCAAGGAUGACUUGCCUG***C***
Vv-miR169i	***U***GAGCCAAGGAUGACUGGCCGU	**∧** GAGCCAAGGAUGACUGGCCGU
Vv-miR169l	***U***GAGCCAAGGAUGACUUGCCGU	**∧** GAGCCAAGGAUGACUUGCCGU
Vv-miR169q	***A***GAGCCAAGGAUGACUUGCCGG	**∧** GAGCCAAGGAUGACUUGCCGG
Vv-miR169r,u	UGAGUCAAGGAUGACUUGCCG***U***	UGAGUCAAGGAUGACUUGCCG**∧**
Vv-miR169t	CGAGUCAAGGAUGACUUGCCG***A***	CGAGUCAAGGAUGACUUGCCG **∧**
Vv-miR169x	UAGCCAAGGAUGACUUGCCU **∧**	UAGCCAAGGAUGACUUGCCU***A***
Vv-miR171c,d	**∧∧∧** UUGAGCCGUGCCAAUAUC***ACG***	**UGA**UUGAGCCGUGCCAAUAUC **∧∧∧**
Vv-miR171i	***U***UGAUUGAGCCGUGCCAAUAUC	**∧** UGAUUGAGCCGUGCCAAUAUC
Vv-miR172d	**∧∧** AGAAUCUUGAUGAUGCUGCAU	***UG***AGAAUCUUGAUGAUGCUGCAU
Vv-miR319b,c,f	***C***UUGGACUGAAGGGAGCUCCC	**∧** UUGGACUGAAGGGAGCUCCCU
Vv-miR319g	***A***UUGGACUGAAGGGAGCUCCC	UUGGACUGAAGGGAGCUCCC***A***
Vv-miR394a,c	UUGGCAUUCUGUCCACCUCC **∧∧**	UUGGCAUUCUGUCCACCUCC***AU***
Vv-miR396b	UUCCACAGCUUUCUUGAACU***U***	UUCCACAGCUUUCUUGAACU **∧**
Vv-miR399b	UGCCAAAGGAGAGUUGCCCU**∧**	UGCCAAAGGAGAGUUGCCCU***G***
Vv-miR477	***AUC***UCCCUCAAAGGCUUCCAA	**∧∧∧** UCCCUCAAAGGCUUCCAA
Vv-miR482	**∧** CUUUCCUACUCCUCCCAUUCC	***U***CUUUCCUACUCCUCCCAUUCC
Vv-miR535a,b,c	UGACAACGAGAGAGAGCACGC***U***	UGACAACGAGAGAGAGCACGC **∧**
Vv-miR156e	UGACAGAGGAGAGUGAGCAC	No
Vv-miR159d	UUUGGACUGAAGGGAGCUCCU	No
Vv-miR160f	UGCCUGGCUCCCUGUAUGCCA	No
Vv-miR169d	CAGCCAAGGAUGACUUGCCGG	No
Vv-miR171k	UUGAUUGAGCCGUGCCAAUAUC	No
Vv-miR396e	UUCCACGGCUUUCUUGAACUU	No
Vv-miR397b	UCAUUGAGUGCAGCGUUGAUG	No
Vv-miR399d	UGCCAAAGGAGAUUUGCUC	No
Vv-miR399e	UGCCAAAGGAGAUUUGCCCGG	No
Vv-miR477a,b,c, f,g	CUCCCUCAAAGGCUUCCA	No
Vv-miR477d	UCCCUCAAAGGCUUCCAA	No
Vv-miR529	AGAAGAGAGAGAGUACAGCU	No
Vv-miR535d,e	UGACAACGAGAGAGAGCACGCU	No
Vv-miR535i	UGACAGCGAGAGAGAGCACAC	No
Vv-miR529	UUAGAUGAUCAUCAACAAAC	No
Vv-miR827	UUAGAUGAUCAUCAACAAAC	No
Vv-miR1030a,b	U**U**UGCAUUUGCACCUGCACCUG	No
Vv-miR171a,b	No	UGAUUGAGCCGUGCCAAUAUC
Vv-miR171g	No	UUGAGCCGAACCAAUAUCACC
Vv-miR171h	No	UGGUUGAGCCGCGCCAAUAUC
Vv-miR395n	No	CUGAAGAGUCUGGAGGAACUC

Notes: The red bases and ∧ represent more or less bases between the Vv-miRNAs sequences from two grapevine cultivars (‘Summer Black’ and ‘Pinot Noir’).

### Prediction of target genes for Vv-miRNAs identified by miR-RACE

Full insight into the miRNA targets will help us to understand the range of miRNAs' regulation on functional genes and to more comprehensively describe the functional importance of these miRNAs [28]–. In this study, a total of 134 potential target genes (Table S2) for 25 Vv-miRNA families could be predicted based on perfect or near perfect matches between miRNAs and their target genes following a set of criteria reported [32], [33]. According to the functional annotation of these potential target genes, they could be classified into three groups, with the largest group being genes encoding transcription factors that could be involved in plant growth, development and phase change from vegetative to reproductive growth (Table S2). The second largest group contained target genes that could encode different enzyme proteins implicated in diverse metabolic processes, such as ATP sulfurylase/APS kinase, ATP synthase, etc. The members of the last group were related to immune response (*NLA*), stress defense (*CWP3*), disease resistance (*NBS-LRR*), and signaling transduction (*ARF, AFB*). The annotated functions of most potential target genes in grapevine were consistent with those identified in *Arabidopsis* [15], *Oryza sativa* [34], *Medicago truncatula* [35], *Lycopersicon esculentum* [36], and *Citrus spp.* [37], indicating that these target genes of orthologous miRNAs in various plant species are functionally conserved. Despite this, there were still some potential target genes that could not be annotated (Table S2), and were thus postulated to be grapevine specific, or were observed due to the incomplete coverage of mRNAs of various plants.

A significant finding in our study is the observation that nucleotide discrepancies in the aligned homologous Vv-miRNAs from various grapevine cultivars could not change their target genes in the cultivars. This is a pointer towards the functional conservation of Vv-miRNAs in the grapevine species. Whether or not the nucleotide discrepancy of orthologous Vv-miRNAs in grapevine and other plants can influence the regulation levels on their target genes can be interesting and important study areas.

### Comparison of targeting patterns of orthologous miRNAs with sequence discrepancy in grapevine and other plants

**Figure 1:**
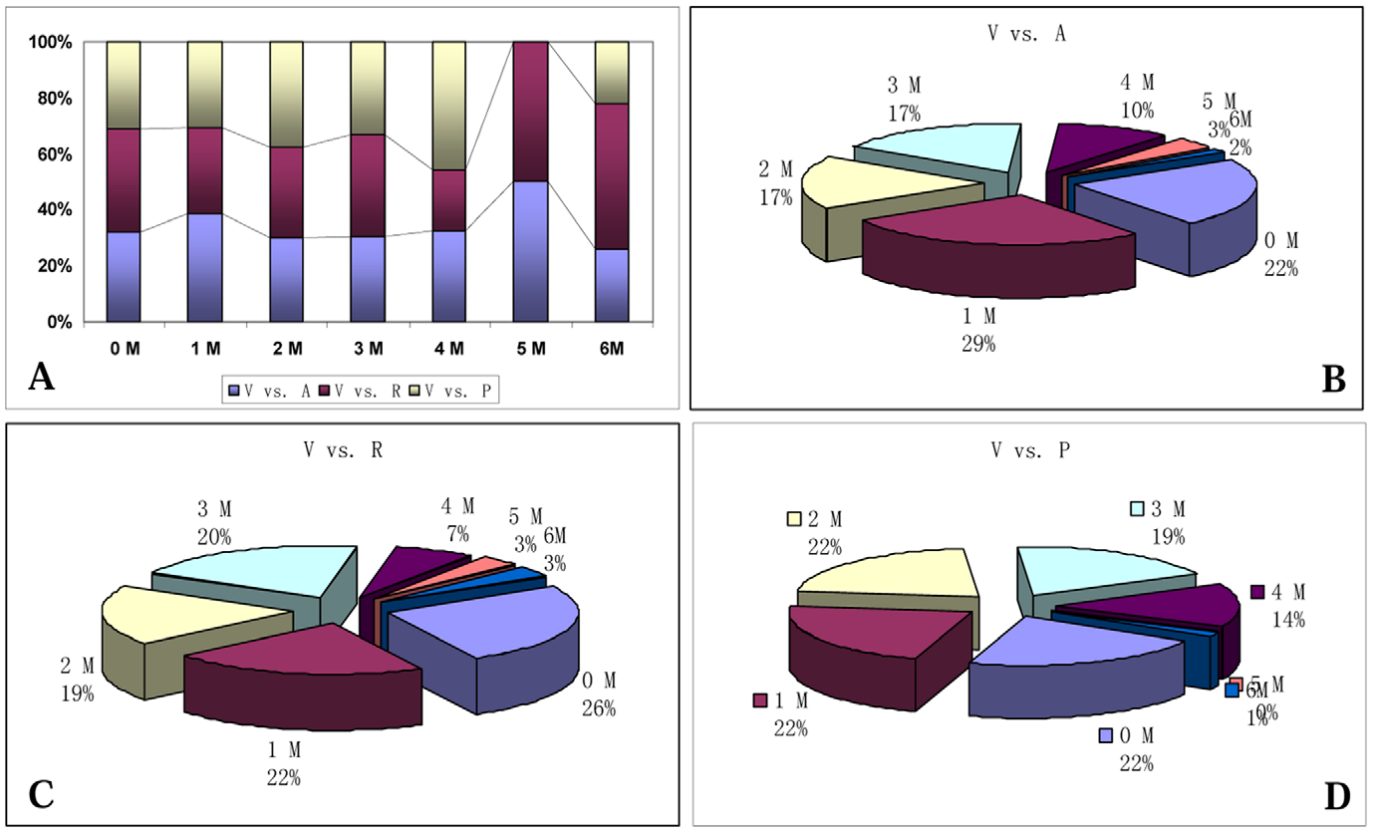
Comparison of divergence of orthologous miRNA sequences among grapevine, *Arabidopsis*, rice and poplar. V vs. A: grapevine miRNA sequences compared to those of *Arabidopsis*; V vs. R: grapevine miRNA sequences compared to those of rice; V vs. P: grapevine miRNA sequences compared to those of poplar.

There have been reports on variation of miRNA sequences among diverse plant species due to evolution [38], [39]. The study on whether the variation of orthologous miRNA sequences in different plants can influence their targeting patterns is an interesting and important research work. Consequently, comparison of orthologous miRNAs' sequence can provide important information for further investigation on the divergence of their target genes in various plants. In this work, the 83 unique Vv-miRNAs' sequences were first aligned with their orthologs in *Arabidopsis*, rice, and poplar (miRbase 15.0 http://www.mirbase.org) [15], [22], [24], [25], [34], [38], where 58, 59, 70 miRNAs in these three model plants could be aligned, respectively. This sequence alignment analysis shows that sequence identity between Vv-miRNAs and their orthologs in *Arabidopsis*, rice, and poplar were in ranges of 72.14%–100%, 70.83%–100%, 74.83%–100%, respectively, demonstrating the sequence variation phenomena of orthologous miRNAs in various plants. Statistically, the identity levels between the miRNA orthologs in grapevine and the 3 plants could be categorized into 7 situations (Fig. 1; Table 2): perfect conservation (0 bases mismatched), 1 base mismatched, 2 bases mismatched, 3 bases mismatched, 4 bases mismatched, 5 bases mismatched, and 6 bases mismatched, whereby the situation with 0 and 1 base mismatched accounted for a percentage of a percentage of 43.7%, indicating the relatively high conservation of miRNAs between grapevine and the three model plants. Nonetheless, the sequence divergence was also obvious. As shown in Fig. 1, the percentage (1.4%) of the Vv-miRNAs being mismatched by 5 and 6 bases with those of poplar was the lowest among the comparison of orthologous miRNAs from grapevine and the above three plants, while the percentage (6.8%) of those mismatched with their orthologues in rice was the highest. The best possible explanation of this phenomenon could be that grapevine and poplar were both dicots and woody plants, whereas rice was a monocot.

**Table 2:** Divergence of experimentally identified orthologous miRNA sequences in four plant species.

Number of mismatched nucleotides (M)	0	1	2	3	4	5	6	total
V vs. A	13	16	10	10	6	2	1	58
V vs. R	15	13	11	12	4	2	2	59
V vs. P	15	15	15	13	10	0	1	69

Notes: V denotes *Vitis vinifera*; A denotes *Arabidopsis*; R denotes Rice; P denotes Poplar; vs: compare.

To systematically understand the targeting patterns of Vv-miRNAs, comparison of the sequences of target genes for a total of 59 pairs of experimentally verified orthologous miRNAs both in grapevine and *Arabidopsis* with 0–6 divergent nucleotides was performed, and the results (Table S3) indicate that the predicted target genes for 30 pairs of orthologous miRNAs were identical or belonged to different members of a same gene family, indicating the target genes regulated by these orthologous miRNAs had high functional conservation both in grapevine and *Arabidopsis*. The target genes for other 16 pairs of orthologous miRNAs could only be predicted in either grapevine or *Arabidopsis* (Table S3). It was also noticed that the target genes of miR397a, miR397b and miR399d were predicted to be vary between grapevine and *Arabidopsis*.

**Figure 2:**
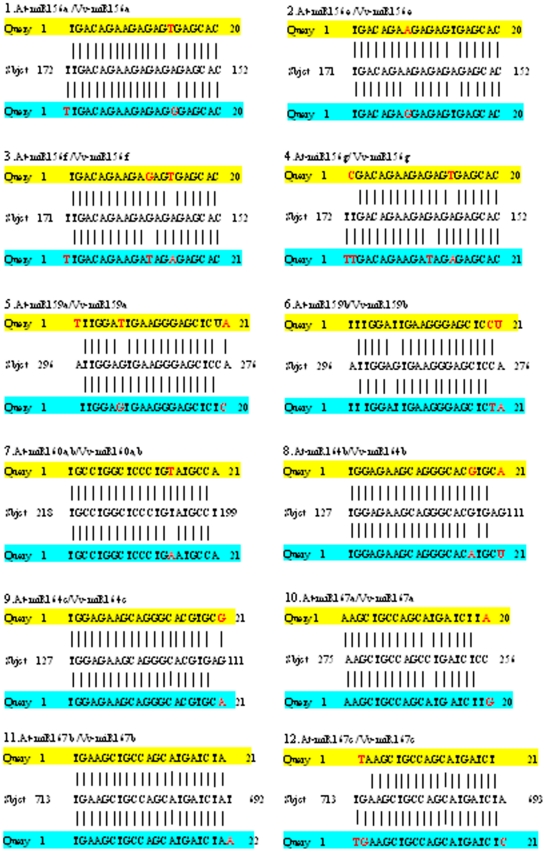
Comparison of the uncomplementary bases of orthologous miRNAs from *Arabidopsis* and grapevine with the same target sequence. The yellow regions are *Arabidopsis* miRNAs sequences, the blue regions are grapevine miRNAs, and the middle sequences are the complementary sequences on target genes of grapevine.

**Figure 3:**
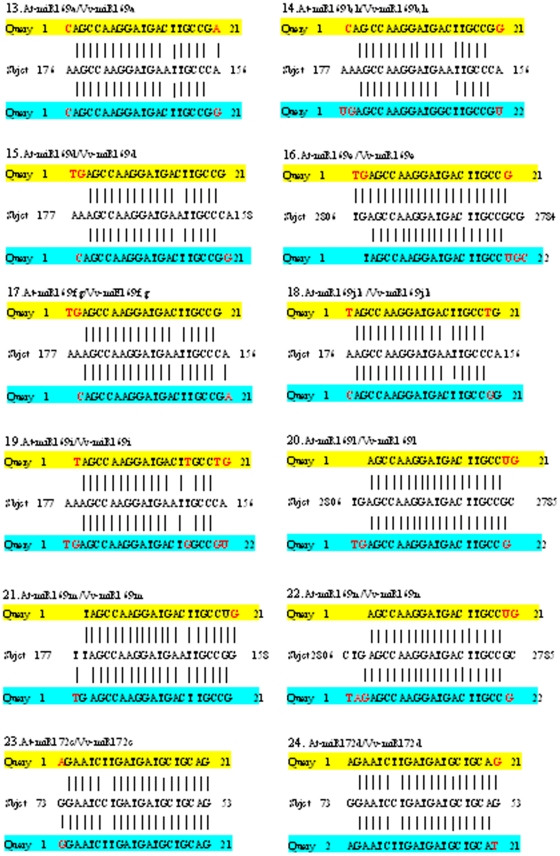
Comparison of the uncomplementary bases of orthologous miRNAs from *Arabidopsis* and grapevine with the same target sequence (continued). The yellow regions are *Arabidopsis* miRNAs sequences, the blue regions are grapevine miRNAs, and the middle sequences are the complementary sequences on target genes of grapevine.

**Figure 4:**
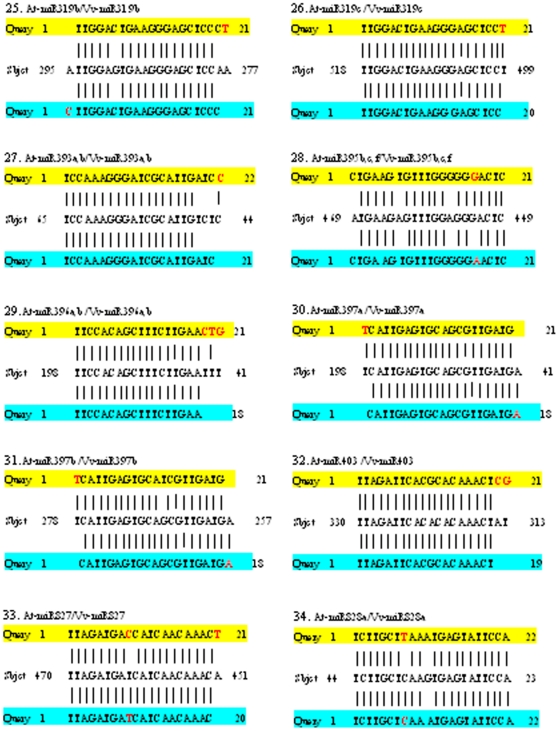
Comparison of the uncomplementary bases of orthologous miRNAs from *Arabidopsis* and grapevine with the same target sequence (continued). The yellow regions are *Arabidopsis* miRNAs sequences, the blue regions are grapevine miRNAs, and the middle sequences are the complementary sequences on target genes of grapevine.

To deeply elucidate the influence of sequence divergence of miRNA orthologs in grapevine and *Arabidopsis* on their targeting patterns, we further analyzed if and how the location of variant base of all the 59 pairs of orthologous miRNAs of grapevine and *Arabidopsis* mentioned above could affect their target genes. The mRNAs search results for all these miRNA orthologs show that 56 pairs of the orthologous miRNAs (belonging to 43 pairs of unique sequences) targeted the same potential genes, even though the complementary sequences in the target genes exhibited some base variation (Fig. 2, 3 and 4, Table 3), suggesting that these orthologous miRNAs were functionally conserved. Based on the difference in the complementary levels of the 43 pairs of unique orthologous sequences with their target genes, they could be classified into four situations (Fig. 2, 3 and 4): (1) sequences of 9 Vv-miRNAs (Vv-miR156b/c/d, Vv-miR160c, Vv-miR164a, Vv-miR169c, Vv-miR172e, Vv-miR394a/b, Vv-miR395a/d/e and Vv-miR408, Vv-miR828a) and their complementary sequences in their target genes were both identical to those in *Arabidopsis*; (2) 7 Vv-miRNAs (Vv-miR167a, Vv-miR167b, Vv-miR169d, Vv-miR169j/k, Vv-miR169i, Vv-miR319b, and Vv-miR403) were different in sequence from their othologs in *Arabidopsis*, but they targeted the same complementary sites on their orthologous target genes; (3) 8 Vv-miRNAs namely Vv-miR156e, Vv-miR160a/b, Vv-miR164b, Vv-miR169b/h, Vv-miR169i, Vv-miR395b/c/f, Vv-miR827, Vv-miR828a and their complementary sequences in their target genes were same in length with their corresponding ones in *Arabidopsis*, though some nucleotides varied in either the sequences of Vv-miRNAs or the complementary sequences; and (4) the sequences of 19 Vv-miRNAs (Vv-miR156a, Vv-miR159a, Vv-miR159b, Vv-miR164a, Vv-miR167c, Vv-miR169a, Vv-miR169d, Vv-miR169e, Vv-miR169f/g, Vv-miR169l, Vv-miR169m, Vv-miR169n, Vv-miR172c, Vv-miR172d, Vv-miR319c, Vv-miR393a/b, Vv-miR396a/b, Vv-miR397a, Vv-miR397b) and their complementary sequences were 1–3 bases more or less in length than their orthologs in *Arabidopsis*. Interestingly, most Vv-miRNA nucleotide variations happened to the mismatched locations between *Arabidopsis* miRNAs (*At*-miRNAs) and their target sequences (Fig. 2, 3 and 4), indicating the mismatched nucleotide sites of miRNAs and their target genes could be the most variable loci, even though these variations did not often change the targeting of these Vv-miRNAs on their target genes.

**Table 3:** Comparison of varied bases of orthologous miRNAs in grapevine and *Arabidopsis* and binding sites of them.

MiRNA	Varied base numbers in miRNA orthologs	Binding sites of varied bases in target sequences	Mismatch number between miRNA and its target	Mismatch number < or = 4?
At-miR156a	2	<$>\raster="rg1"<$>□	1	Y
Vv-miR156a			2	Y
At-miR156e	1	▴	1	Y
Vv-miR156e			2	Y
At-miR156f	3	▵▴<$>\raster="rg1"<$>	1	Y
Vv-miR156f			1	Y
At-miR156g	4	▵▴<$>\raster="rg1"<$>□	2	Y
Vv-miR156g			1	Y
At-miR159a	3	▵<$>\raster="rg1"<$>▪	3	Y
Vv-miR159a			2	Y
At-miR159b	2	□▪▵	3	Y
Vv-miR159b			4	Y
At-miR160a/b	1	▴	1	Y
Vv-miR160a/b			2	Y
At-miR164b	2	□▴	2	Y
Vv-miR164b			3	Y
At-miR164c	1	▪	1	Y
Vv-miR164c			2	Y
At-miR167a	1	□	3	Y
Vv-miR167a			3	Y
At-miR167b	1	<$>\raster="rg1"<$>	0	Y
Vv-miR167b			1	Y
At-miR167c	3	□<$>\raster="rg1"<$>	1	Y
Vv-miR167c			1	Y
At-miR169a	1	▪	3	Y
Vv-miR169a			4	Y
At-miR169b/h	3	□<$>\raster="rg1"<$>	4	Y
Vv-miR169b/h			6	N
At-miR169d	3	□<$>\raster="rg1"<$>	4	Y
Vv-miR169d			4	Y
At-miR169e	5	<$>\raster="rg1"<$>▪	0	Y
Vv-miR169e			4	Y
At-miR169f,g	3	□<$>\raster="rg1"<$>	4	Y
Vv-miR169f,g			3	Y
At-miR169j/k/m	2	□	4	Y
Vv-miR169j/k/m			4	Y
At-miR169i	5	<$>\raster="rg1"<$>□▴	4	Y
Vv-miR169i			6	N
At-miR169l	5	□<$>\raster="rg1"<$>	2	Y
Vv-miR169l			2	Y
At-miR169m	3	▪<$>\raster="rg1"<$>	1	Y
Vv-miR169m			2	Y
At-miR169n	5	<$>\raster="rg1"<$>□	2	Y
Vv-miR169n			3	Y
At-miR172c	1	□	2	Y
Vv-miR172c			1	Y
At-miR172d	1	▪	2	Y
Vv-miR172d			3	Y
At-miR319b	2	<$>\raster="rg1"<$>	3	Y
Vv-miR319b			3	Y
At-miR319c	1	▪	0	Y
Vv-miR319c			0	Y
At-miR393a/b	1	<$>\raster="rg1"<$>	3	Y
Vv-miR393a/b			4	Y
At-miR395b/c/f	1	▴	3	Y
Vv-miR395b/c/f			4	Y
At-miR396a/b	3	<$>\raster="rg1"<$>	2	Y
Vv-miR396a/b			0	Y
At-miR397a	2	<$>\raster="rg1"<$>	0	Y
Vv-miR397a			0	Y
At-miR397b	3	<$>\raster="rg1"<$>▵	1	Y
Vv-miR397b			0	Y
At-miR403	2	<$>\raster="rg1"<$>	2	Y
Vv-miR403			0	Y
At-miR827	2	<$>\raster="rg1"<$>▵	2	Y
Vv-miR827			0	Y
At-miR828a	1	▵	2	Y
Vv-miR828a			1	Y

Notes: At: *Arabidopsis*; Vv: grapevine; Y: Yes; N: No; <$>\raster="rg1"<$>: more or less 1–4 terminal bases of Vv-miRNAs compared to those in *Arabidopsis*; □: variable terminal bases, being mismatched with the corresponding bases of target genes, on orthologous miRNAs both of grapevine and *Arabidopsis*; ▪: varied terminal bases of Vv-miRNAs changing to be mismatched with the corresponding bases of target genes that match to At-miRNAs; ▵: variable inner bases, being mismatched with the corresponding bases of target genes, of orthologous miRNAs ofgrapevine and *Arabidopsis*; ▴: varied inner bases of Vv-miRNAs changing to be mismatched with the corresponding bases of target genes that match to At-miRNAs.

### Expression analysis of miRNAs in grapevine

**Figure 5:**
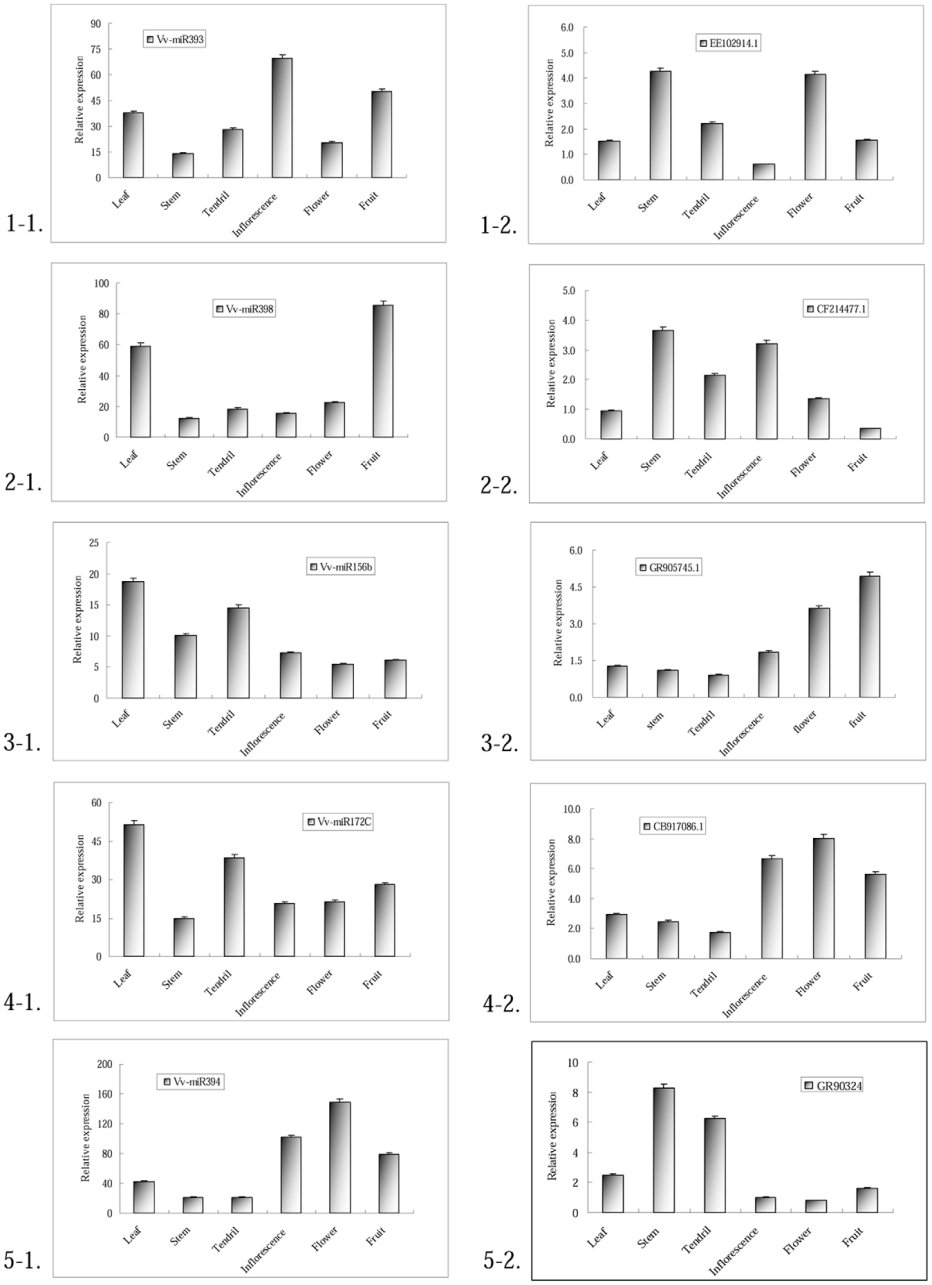
Expression patterns of Vv-miRNAs and their target genes. Lanes: leaf (5 cm in diameter), stem (0.2 cm in diameter), tendril (0.1 cm in diameter), inflorescence (0.2 cm in diameter per grain), flower (0.25 cm in diameter per grain) and berry (1.5 cm in diameter). Each reaction was repeated three times and the template amount was corrected by 5.8 s rRNAs. The left and right graph of each row are the expression patterns of a Vv-miRNA and its target gene.

**Figure 6:**
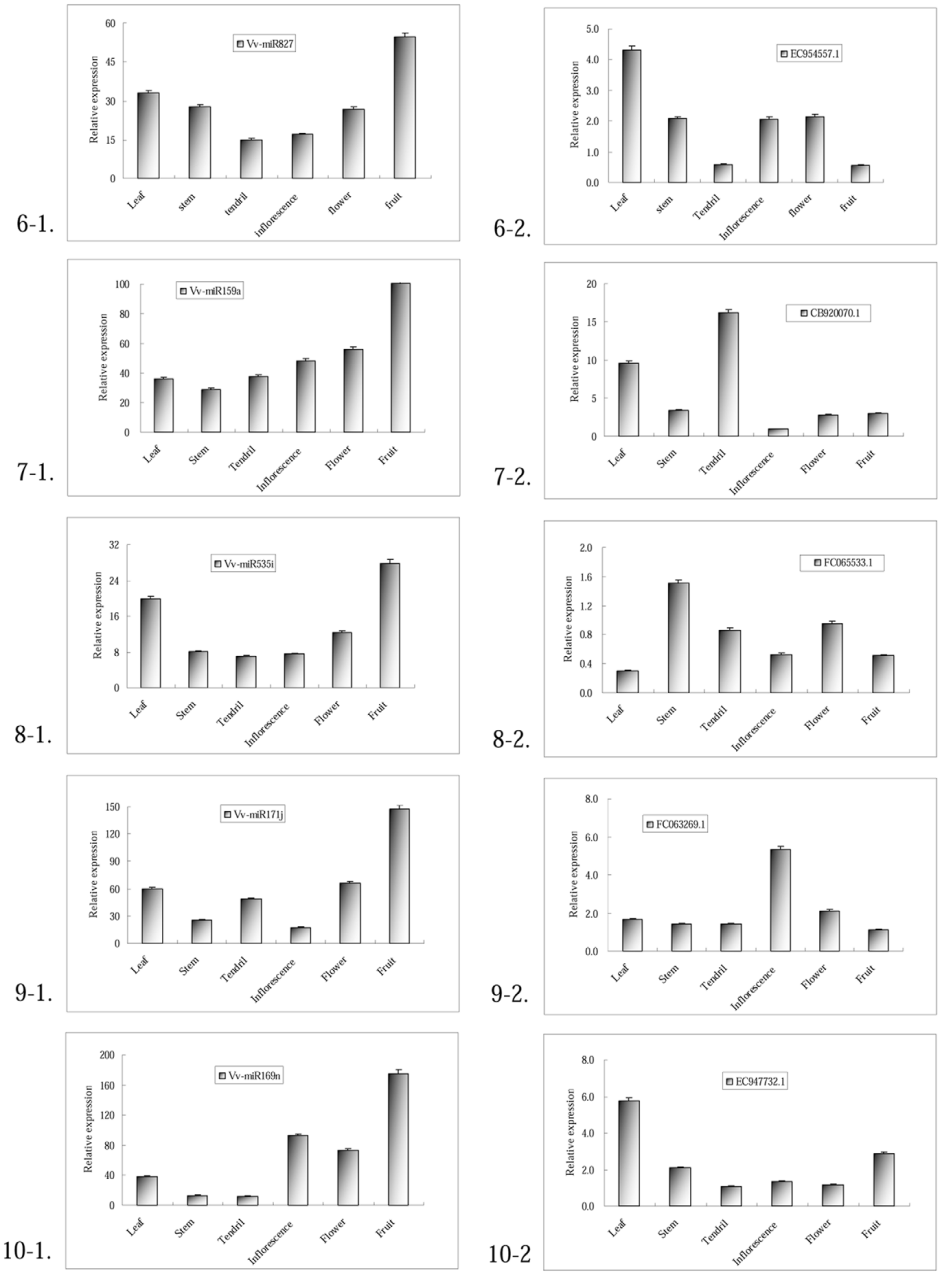
Expression patterns of Vv-miRNAs and their target genes (continued). Lanes: leaf (5 cm in diameter), stem (0.2 cm in diameter), tendril (0.1 cm in diameter), inflorescence (0.2 cm in diameter per grain), flower (0.25 cm in diameter per grain) and berry (1.5 cm in diameter). Each reaction was repeated three times and the template amount was corrected by 5.8 s rRNAs. The left and right graph of each row are the expression patterns of a Vv-miRNA and its target gene.

Preferential expression of miRNAs in specific tissues not only supports the existence of the miRNAs in the organism, but also provides clues on their physiological functions. To further elucidate the Vv-miRNAs regulation pathways, we randomly selected 10 Vv-miRNAs and studied their expressions in leaves, stems, tendril, inflorescences, flowers and fruits of the grapevine cultivar ‘Summer Black’ using qRT-PCR. All the qRT-PCR reaction products were cloned and sequenced for confirmation. As shown in Fig. 5 and 6 (i-1), 10 Vv-miRNAs were expressed at various expression levels in diverse organs, indicating the existence of these potential Vv-miRNAs in grapevine, and the different expression levels could suggest that these Vv-miRNAs were tissue or developmental-stage specific. For example, Vv-miR156b and Vv-miR172c were expressed at higher levels in vegetative tissues (leaf, stem) than those in reproductive organs (inflorescence, flower, young fruit) (Fig. 5 and 6, 3-1, 4-1), with their expression levels in young fruits being similar to those reported by Mica *et al.* [25], while other miRNAs like Vv-miR171j, Vv-miR169 and Vv-miR394 displayed opposite expression patterns in that they had higher expression levels in reproductive organs than they did in vegetative ones (Fig. 5 and 6, 19-1, 10-1, 5-1). Vv-miR398, Vv-miR535i exhibited another kind of expression pattern with strong expression in leaves and fruits and slight expression in the other organs (Fig. 5 and 6, 2-1, 8-1). In addition, some of them exhibited similar holistic expression levels where they were ubiquitously expressed in all tissues and organs. Vv-miR159a, Vv-miR827, Vv-miR394 were highly expressed holistically (Fig. 5 and 6, 7-1, 6-1, 5-1), while Vv-miR156b and Vv-miR535i were lowly expressed holistically (Fig. 5 and 6, 3-1, 8-1). In overall, qRT-PCR analysis could give an outline of the physiological functions of Vv-miRNAs during grapevine growth and development.

### Expression analysis of target genes for Vv-miRNAs

Plant miRNAs play critical gene-regulation roles at the post-transcriptional level mainly through miRNA-directed cleavage and translational inhibition of target genes [33], [40]–[42] in response to environmental stimuli [19], [43] as well as in the plant developmental processes [44]–[46]. The study on the functions of target genes is necessary for elucidation of functions of the corresponding miRNAs. In order to reveal the correlations between Vv-miRNAs and their target genes as well as their interaction mechanisms in controlling grapevine growth and development, we performed quantitative expression analysis of 10 corresponding target genes of the 10 Vv-miRNAs studied in the above section. For this, we used the high molecular weight (HMW) fraction of RNA, taken from the same plants used for miRNA qRT-PCR identification and expression. All of the RT-PCR reaction products were also cloned and sequenced for positive verification. The results (Fig. 5 and 6, i-2) show that the 10 target genes could be detected at various expression levels in diverse tissues, suggesting that these target genes could have divergent functions in various organs and/or function at various levels. As for the expression patterns of these Vv-mRNAs, most exhibited various tissue-specific or developmental-stage specific expression patterns. Good examples are EC954557.1(*Vv-NLA*), CB920070.1 (*Vv-MYB124*), FC065533.1 (*Vv-AEC*) and EC947732.1 (*Vv-NF-YA8*) which were strongly expressed in only one tissue (Fig. 5 and 6, 6-2, 7-2, 8-2, 10-2) and at lower levels in the other tissues. Other target genes such as GR905745.1 (*Vv-SPL9*) and CB917086.1 (*Vv-AP2*) accumulated at higher level only in reproductive organs (Fig. 5 and 6, 3-2, 4-2). Target genes such as EE102914.1 (*Vv-TIR*), CF214477.1 (*Vv-LAC*), EC954557.1 (*Vv-NLA*) and FC065533.1 (*Vv-AEC*) were ubiquitously expressed in all tissues (Fig. 5 and 6, 1-2, 2-2, 6-2, 8-2). Our findings strongly confirm that these target genes could play diverse roles during grapevine growth and development, which is an important contribution towards comprehensive understanding of the regulatory functions of miRNAs.

More importantly, as shown in Fig. 5 and 6, opposite trends in the expressions levels of each of the 10 randomly selected miRNAs and their 10 target genes could be clearly exhibited, indicating that these target genes could be actively cleaved at some levels by equivalent miRNAs, in agreement with the popularly known mode of the regulation by miRNAs on their targets in plants [33], [40]–[42]. A good example of this aspect is the case of EE102914.1 targeted by Vv-miR393 whereby Vv-miR393 had weak expression in grapevine stem and flower, while EE102914.1 had strong expression. Inversely, Vv-miR399 showed a higher level expression in grapevine flower and stem where its target gene was lowly expressed there (Fig. 5 and 6, 1-1, 1-2). Other Vv-miRNAs (Vv-miR156b, Vv-miR172c) had stronger expression level in vegetative organs (leaf and stem), but weaker expression in reproductive organs (inflorescence, flower). On the contrary, their target genes (GR905745.1 and CB917086.1) had weak expression in the equivalent vegetative organs, but had strong expression in the reproductive organs (Fig. 5 and 6, 3-1, 3-2; 4-1, 4-2). In this study, the expression patterns of Vv-miR172c and its target gene were similar with the situations of their orthologs in maize where *GLOSSY15* (*GL15*) was down-regulated by miR172 during vegetative development [28]. The situation of Vv-miR156 was similar with those reported by Yamaguchi *et al.* [30] and Wang *et al.* [31] in *Arabidopsis*. Other target genes like CF214477.1, CB920070.1, EC947732.1, FC063269.1, FC065533.1 had weak expression in grapevine fruit (Fig. 5 and 6, 1-2, 5-2, 8-2, 7-2, 6-2), whereas their corresponding Vv-miRNAs had relatively strong expression in the fruit (Fig. 5 and 6, 1-1, 5-1, 8-1, 7-1, 6-1). Overall, the expression patterns of most of Vv-miRNAs/target gene pairs appeared to be tissue or development stage specific, and ubiquitous expression profiles of these Vv-miRNAs and their target genes exhibited some inverse variation trends between them.

## Discussion

### Validation of precise sequences of Vv-miRNAs from bioinformatics prediction

Grapevine is currently among the fruit crops with the most miRNAs predicted computationally, and the candidate Vv-miRNAs' sequence can be used to design the specific primers both for validation of their precise sequences and discovery of the Vv-miRNAs in a different cultivar by miR-RACE. Currently, quite a number of Vv-miRNAs have been experimentally verified [24], [26], but all of these validated Vv-miRNAs were from different cultivars of the same variety group (*Vitis vinifera* L.), and the orthologous Vv-miRNAs sequenced in the various cultivars showed base discrepancy that only happened at two terminals of the sequences, which was similar to the report in *Arabidopsis* by Felippes *et al.* [39] that some orthologous At-miRNAs from various ecological groups show base discrepancies at either ends of their sequences. These cases suggest that the verification of the termini nucleotides of candidate Vv-miRNAs predicted computationally and those cloned from some grapevine cultivars can be an approach for discovery of true-to-type orthologous miRNAs in different grapevine cultivars. To the best of our knowledge, there was no report on this aspect in a grapevine cultivar. This became the main consideration that triggered initiation of this new research on grapevine cultivar ‘Summer Black’, belonging to another variety group (hybrids of *V. vinifera* and *V. labrusca*) different from those wine grapevine cultivars (*Vitis vinifera* L.) used in the previous studies on Vv-miRNAs. The choice of the candidate Vv-miRNAs predicted computationally to be validated for their precise sequences in cv. ‘Summer Black’ in this study was motivated by the fact that these Vv-miRNAs were of the largest group available publicly, while the choice of employing miR-RACE to validate the sequences was from the consideration that miR-RACE was a powerful method in the validation of termini nucleotides of miRNA predicted [20]. The Vv-miRNAs' sequences validated can provide a solid support for initiation of further studies on miRNA evolution, regulatory roles and biogenesis mechanisms.

So far, miR-RACE can be thought to be the most workable technology employed in identification of full precise sequences of candidate miRNAs [20], [21], indicating it can also be an ideal method of the discovery of true-to-type sequences of miRNAs in a different cultivar as we indicated as above. Compared with previous studies, this technology can not only overcome the shortcomings of the past experimental validation of miRNAs that mainly focused on checking the expression of the miRNAs by Northern blotting and/or RT-PCR both of which only validate the existence and size of miRNAs, but also possess an obvious advantage of the ability to determine the precise sequences of non-abundant miRNAs which are typically difficult to clone directly. Furthermore, miR-RACE technology has higher accuracy, which mainly depends on the design of the two miRNA-specific primers (MSP1, MSP2) of the 3′ and 5′miR- RACE PCRs for precise end sequences of miRNAs. The majortity (96.5%) of all miRNAs predicted computationally in grapevine were exactly identified experimentally here, which confirmed that the number (17) of nucleotides of the miRNA chosen for primer design was very reasonable, consistent with those reported by Song [20], and that a combination of this technology and bioinformatics prediction can be employed to discover a number of miRNAs in various cultivars of grapevine.

### Alignment analysis of Vv-miRNAs sequences from experimental identification and bioinformatics prediction

Among the 83 unique sequences of Vv-miRNAs predicted bioinformatically, 60 were identical to their corresponding sequences cloned by miR-RACE, while other 23 were validated and shown to be 1–4 bases divergent. These results are supported by our deep sequencing of the small RNA library from the same grapevine cultivar, from which 65 corresponding unique sequences of Vv-miRNA sequences were acquired. The possible reasons for the divergence between the cloned and the predicted miRNAs sequences could be explained as a consequence of some error in the miRNAs' prediction due to lack of characteristics defining miRNA at both ends, the evolution of miRNAs and stringency of parameters used in the miRNA predictions, transcription and miRNA processing, as well as use of different grapevine cultivars. In addition, it was also noted that though a large number of Vv-miRNAs could be identified by experimental methods, including deep sequencing of small RNA library, the number and kinds of miRNAs from experimental work were still less than that of miRNAs predicted computationally, which was supported by our sequenced results of high throughput sequencing technology in citrus [37] and grapevine library. All these powerfully confirm that bioinformatics prediction methods have an obvious advantage in massive discovery of miRNAs. Therefore, based on this advantage of bioinformatics approach of miRNA identification together with accurate determination of their precise sequences, especially their termini nucleotides, a large number of miRNAs from various cultivars of grapevine could be discovered, in which an integrated method of the combination of miR-RACE and bioinfomatic prediction technologies were employed. The results in this work powerfully support this view.

### Target genes for Vv-miRNAs

At present, an increasing number of studies indicated that plant miRNAs play key regulatory roles in their growth and development mainly by miRNAs' repressing the expression of target mRNAs through direct cleavage or, in a few cases, by translational repression [40], [47]–[49]. Plant miRNAs, in general, negatively regulate their target genes in various kinds of developmental processes [44]–[46] as well as in response to environmental stimuli [19], [43]. Analysis on the expression of miRNAs' target genes can contribute to elucidation of the functions and the regulation pathway of miRNAs. At the moment, many targets of conserved Vv-miRNA have been predicted [22], [23], and some of them have already been verified experimentally [24], [26], but there are still many target genes without being validated. Importantly, here we also cloned many new Vv-miRNA sequences, of which target genes were not predicted, and discovered some orthologous Vv-miRNAs with variation of termini bases compared with those from other grapevine varieties reported. Some of these with variant termini bases might target diverse genes. Therefore, more systematic prediction of target genes for 83 unique sequences were carried out for further research on target genes' expression and miRNAs' comprehensive functions. A total of 134 target genes, belonging to many important gene families comprising of multiple members, could be predicted here. These families include *MYB*, *GRAS*, *SPB/SPL*, *NAC*, Laccase, *AFB*, ATP sulfurylase ect. It was also shown that a target gene might be regulated by multiple miRNAs and several target genes might be targeted by a single miRNA. These cases indicate there exist some complicated regulatory networks between Vv-miRNAs and their target genes, which can provide plenty of more information for gaining deep and comprehensive insight into Vv-miRNAs functions. In a similar inquiry, Pantaleo *et al.* [24] reports that some target transcripts were regulated by pairs of Vv-miRNAs, such as *SPB* family by miR156 and miR535; *MYB* factor by miR159 and miR319. In this study, we also found some new or different pairs of Vv-miRNA regulating target genes like those reported by Pantaleo *et al.* [24]. For example, *SPB* for Vv-miR156 and Vv-miR529; *MYB* factor for Vv-miR159, Vv-miR319, Vv-miR827 and Vv-miR828; Auxin response factor (*ARF*) for Vv-miR160 and Vv-miR167; *Fbox* for Vv-miR393 and Vv-miR394; GRAS family transcription factor (*GRAS*) for Vv-miR171 and Vv-miR529. This revelation indicates the existence of a more complicated regulating net in grapevines and we expect to explore more and new functions of Vv-miRNAs. Interestingly, new functions of Vv-miR535i that differ from earlier reports [25] were also revealed where Vv-miR535i was predicted to target the gene encoding Auxin efflux carrier family gene (*AEC*), light-harvesting complex I (Lhca1-2) and *Top1* gene in this work. Moreover, as shown in Fig. 2, 3 and 4, Vv-miR535i was highly expressed in the berry, consistent with the report by Mica *et al.* [25], while *AEC* being involved in auxin transportation was down-regulated in berry, exhibiting some opposite variation trend in expression levels, indicating that Vv-miR535 might be involved in the development of grapevine berry by its negatively regulation on *AEC*. Ubiquitous expression profiles of the Vv-miRNAs and their target genes in this study also exhibited negative correlation trends, suggesting that these Vv-miRNAs might negatively regulate their target genes. In addition, the expression patterns of most of them appeared to be tissue or development stage specific, indicating that these Vv-miRNAs play diverse regulatory roles on their target genes in different stages of growth and development in grapevines. All our findings can assist in more comprehensive understanding of the functions of Vv-miRNAs.

## Materials and Methods

### Plant material

Leaves, stems (1 cm in diameter), tendrils, inflorescences, flowers, and developing fruits (20 d after full blooming) were collected from four-year old ‘Summer Black’ grapevine (hybrids of *V. vinifera* and *V. labrusca*) trees grown under standard grapevine cultivation conditions at the Fruit Experimental Farm, Nanjing Agricultural University, Nanjing, China in 2009. After collection, all the samples were immediately frozen in liquid nitrogen and stored at −80°C until being used.

### Low molecular weight RNA extraction

After total RNA was isolated from 200 mg of the selected plant tissues using the CTAB method [50], 10 M LiCl was used to separate the low and larger molecular weight RNA following the procedures reported earlier [51]. The low molecular weight (LMW) RNA fraction was then dissolved in 30 µl of RNase free water and the concentration of the RNA samples was measured by a UV-1800 spectrophotometer (Shimadzu, Japan) and visually ascertained in a 2.5% agarose gel. The larger molecular weight RNA samples were used to study the expression patterns of the target genes of Vv-miRNAs.

### Construction and screening of cDNA libraries of small RNAs

We generated the miRNA-enriched library that has been popularly used to clone miRNAs and to measure the expression of miRNAs via RT-PCR [52], [53], in which 5′- and 3′-end adaptors were linked to the miRNA molecules [20], which were further reverse transcribed using Superscript III reverse transcriptase (Invitrogen) in the presence of random nonamers (Sigma), according to the protocols provided by the manufacturers. After the preparation of miRNA libraries from various organs and tissues, we pooled similar quantities of these library samples for further PCR amplification reactions.

### Analyses of miRNA by 5′miR-RACE and 3′miR-RACE

The cDNA was amplified with the mirRacer 5′ primer (5′- GGACACTGACATGGACTGAAGGAGTA-3′) and the mirRacer 3′ primer (5′-ATTCTAGAGGCCGAGGCGGCCGACATG-3′) to generate a pool of non-gene-specific product. 5′ miR-RACE reactions were performed with the mirRacer 5′ primer and miRNA-gene-specific forward primers (MSP1) (Table S4), and 3′ miR-RACE reactions were carried out with the mirRacer 3′ primer and miRNA-gene-specific reverse primers (MSP2) (Table S4), as described by Song et al. [20], with minor modifications. The 5′ RACE and 3′ RACE clones with PCR products of about 56 bp and 87 bp, respectively, were sequenced (Invitrogen).

### Real-time PCR of miRNAs and their target genes

The template used for RT-PCR was the miRNA-enriched library mentioned above. RT-PCR was conducted with the fluorescence quantitative polymerase real-time quantitative PCR (Bio-Rad) and the Rotor-Gene software version 6.1 [54]. SYBR green reaction mix (SYBR® Green qRT-PCR Master Mix; Toyobo, Osaka, Japan) were used in Real-time PCR reactions, according to the manufacturer's instructions. The CT values were converted into relative copy numbers using a standard curve [55]. The 5.8S rRNA was used as a reference gene in the qPCR detection of miRNAs following the work in *Arabidopsis* [56]. Data were analyzed with an R^2^ above 0.998 using the LinRegPCR program [57]. The primers used are listed in Tables S5 and S6.

### Prediction of potential targets of miRNAs and their functions

Target predictions were performed based on a set of the criteria for predicating targets proposed by Schwab *et al.* [27]. Blast searches of mRNA database for plants were used to successfully select potential miRNA targets in mRNA sequences to predict newly identified miRNA targets in *Vitis vinifera* [16], [58], while BlastX was conducted for the potential functional analysis of their target genes.

### Software employed

The comparative software (BLAST-2.2.14) was downloaded from NCBI GenBank and BLASTX from the web site http://www.ncbi.nlm.nih.gov/BLAST/ used for analysis of potential targets [14]. Putative Vv-miRNAs were first blasted against the grapevine unigene database. BlastN hits with fewer than four nucleotides mismatches (plus/minus) were selected as the candidate targets, which were then searched in Genescope using BlastX to obtain their putative functions [http://www.genoscope.cns.fr/externe/GenomeBrowser/Vitis/].

### Oligonucleotide synthesis and preparation

All the oligonucleotides used were purchased from Invitrogen Technologies, and then purified by desalting. All primers used in this study are as listed in Tables S4, S5, and S6.

## Supporting Information

Figure S1 **3′RACE and 5′ RACE products of Vv-miRNAs amplified by PCR shown in an ethidium bromide-stained agarose gel.** Sizes of the molecular weight markers of the bottom and the second bottom bands are 50 bp and 100 bp, respectively. Lanes 1–8 are 3′RACE (up) and 5′RACE (down) products of Vv-miR156a, Vv-miR160b, Vv-miR164d, Vv-miR393a, Vv-miR397ba, Vv-miR403, Vv-miR482 and Vv-miR535i, respectively. The sizes of 3′RACE products are about 83 bp while the size of 5′RACE products are about 57 bp.
(TIF)

Table S1 **Comparison of Vv-miRNA sequences predicated** ***in silico*** **and the validated by miR-RACE.**
(DOC)

Table S2 **Predicted target genes or proteins for grapevine miRNAs validated by miR-RACE.**
(DOC)

Table S3 **Comparison of target genes of experimentally verified orthologous miRANs in grapevine and** ***Arabidopsis*** **.**
(DOC)

Table S4 **Primers used for miR-5′RACE, miR-3′RACE.**
(DOC)

Table S5 **Primers used for real-time PCR of Vv-miRNAs.**
(DOC)

Table S6 **Primers used for real-time PCR of target genes.**
(DOC)
