# Supplementary figures and images for: Characterization of microRNAs Identified in a Table Grapevine Cultivar with Validation of Computationally Predicted Grapevine miRNAs by miR-RACE
Source: PLoS One. 2011 Jul 28;6(7):e21259. doi: 10.1371/journal.pone.0021259 (PMC3145640; doi:10.1371/journal.pone.0021259)

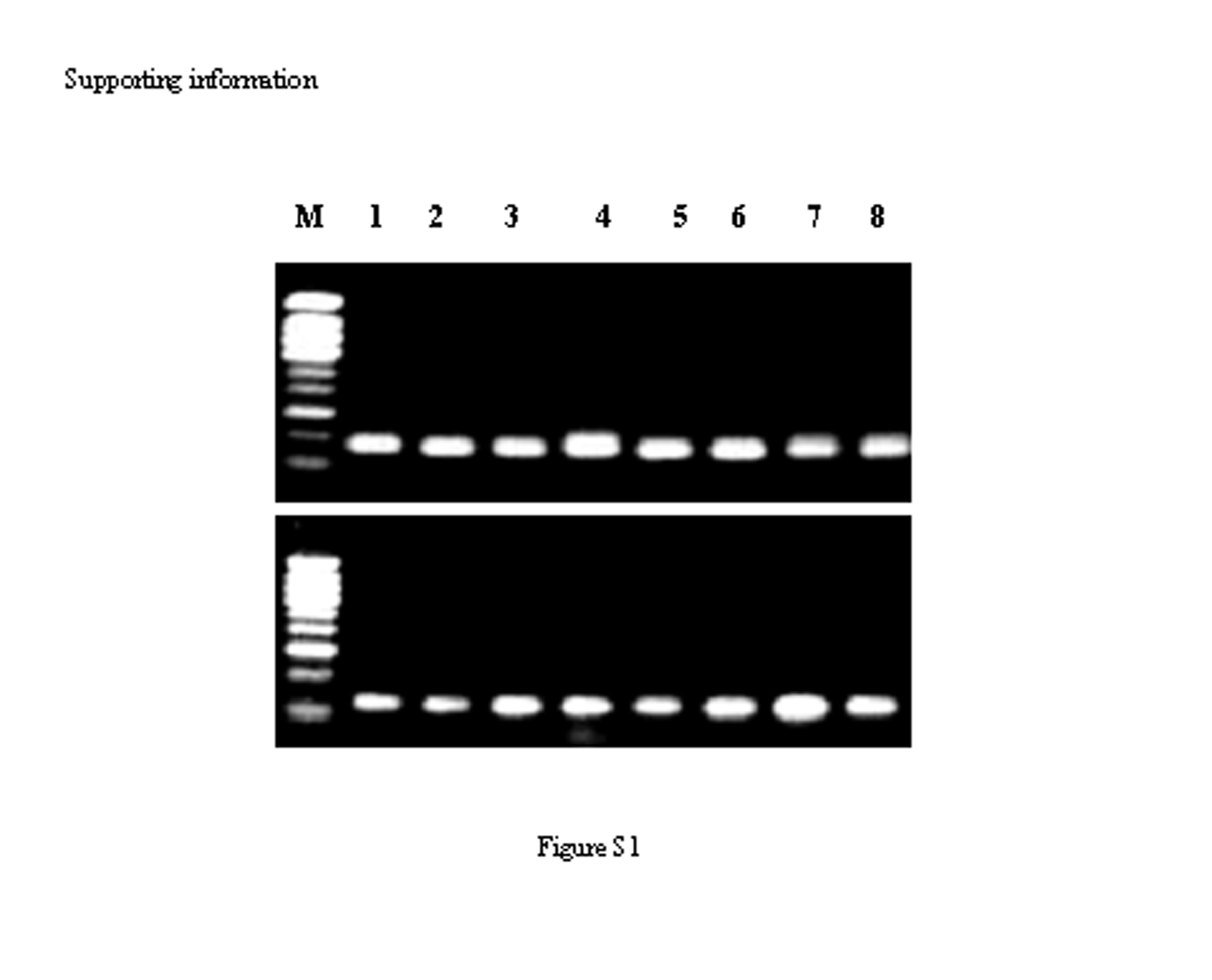

Supplement: Figure S1 — 3′RACE and 5′ RACE products of Vv-miRNAs amplified by PCR shown in an ethidium bromide-stained agarose gel. Sizes of the molecular weight markers of the bottom and the second bottom bands are 50 bp and 100 bp, respectively. Lanes 1–8 are 3′RACE (up) and 5′RACE (down) products of Vv-miR156a, Vv-miR160b, Vv-miR164d, Vv-miR393a, Vv-miR397ba, Vv-miR403, Vv-miR482 and Vv-miR535i, respectively. The sizes of 3′RACE products are about 83 bp while the size of 5′RACE products are about 57 bp. (TIF) [file pone.0021259.s001.tif]
