# Supplementary material for: Characterization of microRNAs Identified in a Table Grapevine Cultivar with Validation of Computationally Predicted Grapevine miRNAs by miR-RACE
Source: PLoS One. 2011 Jul 28;6(7):e21259. doi: 10.1371/journal.pone.0021259 (PMC3145640; doi:10.1371/journal.pone.0021259)
Supplement: Table S1 — Comparison of Vv-miRNA sequences predicated in silico and the validated by miR-RACE. (DOC) [file pone.0021259.s002.doc]

| **Table S1** | | | |
| --- | --- | --- | --- |
| **Grapevine miRNAs** | **Sequences predicated *in silico*** | **Sequences validated by miR-RACE** | **References for predicated sequences** |
| Vv-miR156a | UGACAGAAGAGAGGGAGCAC | UUGACAGAAGAGAGGGAGCAC | Jaillon *et al.*, 2007 |
| Vv-miR156f | UUGACAGAAGAUAGAGAGCAC | UUGACAGAAGAUAGAGAGCAC | Jaillon *et al.* , 2007 |
| Vv-miR156g | UUGACAGAAGAUAGAGAGCAC | UUGACAGAAGAUAGAGAGCAC | Jaillon *et al.*, 2007 |
| Vv-miR156i | UGACAGAAGAUAGAGAGCAC | UUGACAGAAGAUAGAGAGCAC | Jaillon *et al.*, 2007 |
| Vv-miR156b | UGACAGAAGAGAGUGAGCAC | UGACAGAAGAGAGUGAGCAC | Jaillon *et al.*, 2007 |
| Vv-miR156c | UGACAGAAGAGAGUGAGCAC | UGACAGAAGAGAGUGAGCAC | Jaillon *et al.*, 2007 |
| Vv-miR156d | UGACAGAAGAGAGUGAGCAC | UGACAGAAGAGAGUGAGCAC | Jaillon *et al.*, 2007 |
| Vv-miR156e | UGACAGAGGAGAGUGAGCAC | UGACAGAGGAGAGUGAGCAC | Jaillon *et al.*, 2007 |
| Vv-miR156h | UGACAGAAGAGAGAGAGCAU | UGACAGAAGAGAGAGAGC | Jaillon *et al.*, 2007 |
| Vv-miR159a | CUUGGAGUGAAGGGAGCUCUC | UUGGAGUGAAGGGAGCUCUC | Jaillon *et al.*, 2007 |
| Vv-miR159b | CUUGGAGUGAAGGGAGCUCUC | UUGGAGUGAAGGGAGCUCUC | Jaillon *et al.*, 2007 |
| Vv-miR159c | UUUGGAUUGAAGGGAGCUCUA | UUUGGAUUGAAGGGAGCUCUA | Jaillon *et al.*, 2007 |
| Vv-miR159d | UUUGGACUGAAGGGAGCUCCU | UUUGGACUGAAGGGAGCUCCU | Cai *et al.*, 2008 |
| Vv-miR159e | AUUGGACUGAAGGGAGCUCUA | AUUGGACUGAAGGGAGCUCUA | Cai *et al.*, 2008 |
| Vv-miR160a | UGCCUGGCUCCCUGAAUGCCA | UGCCUGGCUCCCUGAAUGCCA | Jaillon *et al.*, 2007 |
| Vv-miR160b | UGCCUGGCUCCCUGAAUGCCA | UGCCUGGCUCCCUGAAUGCCA | Jaillon *et al.*, 2007 |
| Vv-miR160e | UGCCUGGCUCCCUGAAUGCCA | UGCCUGGCUCCCUGAAUGCCA | Jaillon *et al.*, 2007 |
| Vv-miR160c | UGCCUGGCUCCCUGUAUGCCA | UGCCUGGCUCCCUGUAUGCCA | Jaillon *et al.*, 2007 |
| Vv-miR160d | UGCCUGGCUCCCUGUAUGCCA | UGCCUGGCUCCCUGUAUGCCA | Jaillon *et al.*, 2007 |
| Vv-miR160f | UGCCUGGCUCCCUGUAUGCCA | UGCCUGGCUCCCUGUAUGCCA | Jaillon *et al.*, 2007 |
| Vv-miR162 | UCGAUAAACCUCUGCAUCCAG | UCGAUAAACCUCUGCAUCCAG | Jaillon *et al.*, 2007 |
| Vv-miR164a | UGGAGAAGCAGGGCACGUGCA | UGGAGAAGCAGGGCACGUGCA | Jaillon *et al.*, 2007 |
| Vv-miR164c | UGGAGAAGCAGGGCACGUGCA | UGGAGAAGCAGGGCACGUGCA | Jaillon *et al.*, 2007 |
| Vv-miR164d | UGGAGAAGCAGGGCACGUGCA | UGGAGAAGCAGGGCACGUGCA | Jaillon *et al.*, 2007 |
| Vv-miR164b | UGGAGAAGCAGGGCACAUGCU | UGGAGAAGCAGGGCACAUGCU | Jaillon *et al.*, 2007 |
| Vv-miR166a | UCGGACCAGGCUUCAUUCCUG | UCUCGGACCAGGCUUCAUUCC | Jaillon *et al.*, 2007 |
| Vv-miR166b | UCGGACCAGGCUUCAUUCCUC | UCGGACCAGGCUUCAUUCCUC | Jaillon *et al.*, 2007 |
| Vv-miR166c | UCGGACCAGGCUUCAUUCCCCC | UCGGACCAGGCUUCAUUCCCC | Jaillon *et al.*, 2007 |
| Vv-miR166e | UCGGACCAGGCUUCAUUCCCCC | UCGGACCAGGCUUCAUUCCCC | Jaillon *et al.*, 2007 |
| Vv-miR166h | UCGGACCAGGCUUCAUUCCCCC | UCGGACCAGGCUUCAUUCCCC | Jaillon *et al.*, 2007 |
| Vv-miR166d | UCGGACCAGGCUUCAUUCCCCU | UCGGACCAGGCUUCAUUCCCC | Jaillon *et al.*, 2007 |
| Vv-miR166g | UCGGACCAGGCUUCAUUCCCCU | UCGGACCAGGCUUCAUUCCCC | Jaillon *et al.*, 2007 |
| Vv-miR166f | UCGGACCAGGCUUCAUUCCCCU | UCGGACCAGGCUUCAUUCCCC | Jaillon *et al.*, 2007 |
| Vv-miR167a | UGAAGCUGCCAGCAUGAUCUG | UGAAGCUGCCAGCAUGAUCUG | Jaillon *et al.*, 2007 |
| Vv-miR167b | UGAAGCUGCCAGCAUGAUCUA | UGAAGCUGCCAGCAUGAUCUAA | Jaillon *et al.*, 2007 |
| Vv-miR167e | UGAAGCUGCCAGCAUGAUCUA | UGAAGCUGCCAGCAUGAUCUAA | Jaillon *et al.*, 2007 |
| Vv-miR167d | UGAAGCUGCCAGCAUGAUCUA | UGAAGCUGCCAGCAUGAUCUA | Jaillon *et al.*, 2007 |
| Vv-miR167c | UGAAGCUGCCAGCAUGAUCUC | UGAAGCUGCCAGCAUGAUCUC | Jaillon *et al.*, 2007 |
| Vv-miR168 | UCGCUUGGUGCAGGUCGGGAA | UCGCUUGGUGCAGGUCGGGAA | Jaillon *et al.*, 2007 |
| Vv-miR169a | CAGCCAAGGAUGACUUGCCGG | CAGCCAAGGAUGACUUGCCGG | Jaillon *et al.*, 2007 |
| Vv-miR169c | CAGCCAAGGAUGACUUGCCGG | CAGCCAAGGAUGACUUGCCGG | Jaillon *et al.*, 2007 |
| Vv-miR169d | CAGCCAAGAAUGAUUUGCCGG | CAGCCAAGAAUGAUUUGCCGG | Jaillon *et al.*, 2007 |
| Vv-miR169b | AAGCCAAGGAUGAAUUGCCGG | UGAGCCAAGGAUGGCUUGCCGU | Cai *et al.,* 2008 |
| Vv-miR169b | UGAGCCAAGGAUGGCUUGCCG | UGAGCCAAGGAUGGCUUGCCGU | Jaillon *et al.,* 2007 |
| Vv-miR169h | UGAGCCAAGGAUGGCUUGCCG | UGAGCCAAGGAUGGCUUGCCGU | Jaillon *et al.*, 2007 |
| Vv-miR169h | GAGCCAAGGAUGGCUUGCCGU | UGAGCCAAGGAUGGCUUGCCGU | Cai *et al.,* 2008 |
| Vv-miR169i | GAGCCAAGGAUGACUGGCCGU | UGAGCCAAGGAUGACUGGCCGU | Jaillon *et al.*, 2007/Cai *et al.,* 2008 |
| Vv-miR169e | UAGCCAAGGAUGACUUGCCUG | UAGCCAAGGAUGACUUGCCUGC | Jaillon *et al.*, 2007 |
| Vv-miR169f | CAGCCAAGGAUGACUUGCCGA | CAGCCAAGGAUGACUUGCCGA | Jaillon *et al.*, 2007 |
| Vv-miR169g | CAGCCAAGGAUGACUUGCCGA | CAGCCAAGGAUGACUUGCCGA | Jaillon *et al.*, 2007 |
| Vv-miR169j | CAGCCAAGGAUGACUUGCCGG | CAGCCAAGGAUGACUUGCCGG | Jaillon *et al.*, 2007 |
| Vv-miR169k | CAGCCAAGGAUGACUUGCCGG | CAGCCAAGGAUGACUUGCCGG | Jaillon *et al.*, 2007 |
| Vv-miR169s | CAGCCAAGGAUGACUUGCCGG | CAGCCAAGGAUGACUUGCCGG | Jaillon *et al.*, 2007 |
| Vv-miR169w | CAGCCAAGGAUGACUUGCCGG | CAGCCAAGGAUGACUUGCCGG | Jaillon *et al.*, 2007 |
| Vv-miR169w | GAGCCAAGGAUGACUUGCCGG | CAGCCAAGGAUGACUUGCCGG | Cai *et al.,* 2008 |
| Vv-miR169l | UGAGCCAAGGAUGACUUGCCG | UGAGCCAAGGAUGACUUGCCG | Jaillon *et al.*, 2007 |
| Vv-miR169m | CAGCCAAGGAUGACUUGCCGG | UGAGCCAAGGAUGACUUGCCG | Jaillon *et al.*, 2007 |
| Vv-miR169o | UGAGCCAAGGAUGACUUGCCG | UGAGCCAAGGAUGACUUGCCG | Cai *et al ,* 2008 |
| Vv-miR169o | GAGCCAAGGAUGACUUGCCGC | UGAGCCAAGGAUGACUUGCCG | Jaillon *et al.*, 2007 |
| Vv-miR169p | CAGCCAAGGAUGACUUGCCGG | UGAGCCAAGGAUGACUUGCCG | Jaillon *et al.*, 2007 |
| Vv-miR169n | GAGCCAAGGAUGACUUGCCGG | UAGAGCCAAGGAUGACUUGCCG | Jaillon *et al.*, 2007/Cai |
| Vv-miR169q | GAGCCAAGGAUGACUUGCCGG | UAGAGCCAAGGAUGACUUGCCG | Jaillon *et al.*, 2007 |
| Vv-miR169q | UGAGCCAAGGAUGGCUUGCCG | UAGAGCCAAGGAUGACUUGCCG | Cai *et al.,* 2008 |
| Vv-miR169r | UGAGUCAAGGAUGACUUGCCG | UGAGUCAAGGAUGACUUGCCGA | Jaillon *et al.*, 2007/Cai *et al.,* 2008 |
| Vv-miR169u | UGAGUCAAGGAUGACUUGCCG | UGAGUCAAGGAUGACUUGCCGU | Jaillon *et al.*, 2007 |
| Vv-miR169t | CGAGUCAAGGAUGACUUGCCG | CGAGUCAAGGAUGACUUGCCGA | Jaillon *et al.*, 2007 |
| Vv-miR169v | AAGCCAAGGAUGAAUUGCCGG | AAGCCAAGGAUGAAUUGCCGG | Jaillon *et al.*, 2007 |
| Vv-miR169v | UGAGCCAAGGAUGACUUGCCG | AAGCCAAGGAUGAAUUGCCGG | Cai *et al.,* 2008 |
| Vv-miR169x | UAGCCAAGGAUGACUUGCCU**A** | UAGCCAAGGAUGACUUGCCU | Jaillon *et al.*, 2007/Cai *et al.,* 2008 |
| Vv-miR169y | UAGCGAAGGAUGACUUGCCUA | UAGCGAAGGAUGACUUGCCUA | Jaillon *et al.*, 2007 |
| Vv-miR171a | UUGAGCCGUGCCAAUAUCAUG | UGAUUGAGCCGUGCCAAUAUC | Jaillon *et al.*, 2007 |
| Vv-miR171c | UUGAGCCGUGCCAAUAUCACG | UGAUUGAGCCGUGCCAAUAUC | Jaillon *et al.*, 2007 |
| Vv-miR171d | UUGAGCCGUGCCAAUAUCACG | UGAUUGAGCCGUGCCAAUAUC | Jaillon *et al.*, 2007 |
| Vv-miR171b | UGAUUGAGCCGCGUCAAUAUC | UUGAGCCGCGUCAAUAUCUCC | Jaillon *et al.*, 2007 |
| Vv-miR171e | UGAUUGAGCCGCGCCAAUAUC | UUGAGCCGCGCCAAUAUCACU | Jaillon *et al.*, 2007 |
| Vv-miR171f | UUGAGCCGCGCCAAUAUCACU | UUGAGCCGCGCCAAUAUCACU | Jaillon *et al.*, 2007 |
| Vv-miR171g | UUGAGCCGAACCAAUAUCACC | UUGAGCCGAACCAAUAUCACC | Jaillon *et al.*, 2007 |
| Vv-miR171h | UGGUUGAGCCGCGCCAAUAUC | UUGAGCCGCGCCAAUAUCCCG | Jaillon *et al.*, 2007 |
| Vv-miR171i | UUGAGCCGUGCCAAUAUCAUC | UGAUUGAGCCGUGCCAAUAUC | Jaillon *et al.*, 2007 |
| Vv-miR171j | UUGAUUGAGCCGUGCCAAUAUC | UUGAUUGAGCCGUGCCAAUAUC | Cai *et al.*, 2008 |
| Vv-miR171k | UUGAUUGAGCCGUGCCAAUAUC | UUGAUUGAGCCGUGCCAAUAUC | Cai *et al.*, 2008 |
| Vv-miR172c | GGAAUCUUGAUGAUGCUGCAG | GGAAUCUUGAUGAUGCUGCAG | Jaillon *et al.*, 2007 |
| Vv-miR172d | AGAAUCUUGAUGAUGCUGCAU | AGAAUCUUGAUGAUGCUGCAU | Jaillon *et al.*, 2007 |
| Vv-miR172e | UGAAUCUUGAUGAUGCUCCAU | UGAAUCUUGAUGAUGCUCCAU | Cai *et al.*, 2008 |
| Vv-miR319b | CUUGGACUGAAGGGAGCUCCC | UUGGACUGAAGGGAGCUCCC | Jaillon *et al.*, 2007 |
| Vv-miR319c | CUUGGACUGAAGGGAGCUCCC | UUGGACUGAAGGGAGCUCCC | Jaillon *et al.*, 2007 |
| Vv-miR319f | CUUGGACUGAAGGGAGCUCCC | UUGGACUGAAGGGAGCUCCC | Jaillon *et al.*, 2007 |
| Vv-miR319e | UUUGGACUGAAGGGAGCUCCU | UUUGGACUGAAGGGAGCUCCU | Jaillon *et al.*, 2007 |
| Vv-miR319g | AUUGGACUGAAGGGAGCUCCC | UUGGACUGAAGGGAGCUCCC | Jaillon *et al.*, 2007 |
| Vv-miR390/390a | AAGCUCAGGAGGGAUAGCGCC | AAGCUCAGGAGGGAUAGCGCC | Jaillon *et al.*, 2007/Cai *et al.,* 2008 |
| Vv-miR390b | AAGCUCAGGAGGGAUAGCGCC | AAGCUCAGGAGGGAUAGCGCC | Cai *et al.*, 2008 |
| Vv-miR393a | UCCAAAGGGAUCGCAUUGAUC**C** | UUCCAAAGGGAUCGCAUUGAU | Jaillon *et al.*, 2007 |
| Vv-miR393b | UCCAAAGGGAUCGCAUUGAUCC | UCCAAAGGGAUCGCAUUGAU | Jaillon *et al.*, 2007 |
| Vv-miR393b | UCCAAAGGGAUCGCAUUGAUCCC | UCCAAAGGGAUCGCAUUGAU | Cai *et al.,* 2008 |
| Vv-miR394a | UUGGCAUUCUGUCCACCUCC | UUGGCAUUCUGUCCACCUCC | Jaillon *et al.*, 2007 |
| Vv-miR394b | UUGGCAUUCUGUCCACCUCC | UUGGCAUUCUGUCCACCUCC | Jaillon *et al.*, 2007 |
| Vv-miR394c | UUGGCAUUCUGUCCACCUCC | UUGGCAUUCUGUCCACCUCC | Jaillon *et al.*, 2007/Cai *et al.,* 2008 |
| Vv-miR395a | CUGAAGUGUUUGGGGGAACUC | CUGAAGUGUUUGGGGGAACUC | Jaillon *et al.*, 2007 |
| Vv-miR395b | CUGAAGUGUUUGGGGGAACUC | CUGAAGUGUUUGGGGGAACUC | Jaillon *et al.*, 2007 |
| Vv-miR395c | CUGAAGUGUUUGGGGGAACUC | CUGAAGUGUUUGGGGGAACUC | Jaillon *et al.*, 2007 |
| Vv-miR395d | CUGAAGUGUUUGGGGGAACUC | CUGAAGUGUUUGGGGGAACUC | Jaillon *et al.*, 2007 |
| Vv-miR395e | CUGAAGUGUUUGGGGGAACUC | CUGAAGUGUUUGGGGGAACUC | Jaillon *et al.*, 2007 |
| Vv-miR395f | CUGAAGUGUUUGGGGGAACUC | CUGAAGUGUUUGGGGGAACUC | Jaillon *et al.*, 2007 |
| Vv-miR395g | CUGAAGUGUUUGGGGGAACUC | CUGAAGUGUUUGGGGGAACUC | Jaillon *et al.*, 2007 |
| Vv-miR395h | CUGAAGUGUUUGGGGGAACUC | CUGAAGUGUUUGGGGGAACUC | Jaillon *et al.*, 2007 |
| Vv-miR395i | CUGAAGUGUUUGGGGGAACUC | CUGAAGUGUUUGGGGGAACUC | Jaillon *et al.*, 2007 |
| Vv-miR395j | CUGAAGUGUUUGGGGGAACUC | CUGAAGUGUUUGGGGGAACUC | Jaillon *et al.*, 2007 |
| Vv-miR395k | CUGAAGUGUUUGGGGGAACUC | CUGAAGUGUUUGGGGGAACUC | Jaillon *et al.*, 2007 |
| Vv-miR395l | CUGAAGUGUUUGGGGGAACUC | CUGAAGUGUUUGGGGGAACUC | Jaillon *et al.*, 2007 |
| Vv-miR395m | CUGAAGUGUUUGGGGGAACUC | CUGAAGUGUUUGGGGGAACUC | Jaillon *et al.*, 2007 |
| Vv-miR396a | UUCCACAGCUUUCUUGAACUA | UUCCACAGCUUUCUUGAA | Jaillon *et al.*, 2007 |
| Vv-miR396b | UUCCACAGCUUUCUUGAACU^ | UUCCACAGCUUUCUUGAA | Jaillon *et al.*, 2007 |
| Vv-miR396c | UUCCACAGCUUUCUUGAACUG | UUCCACAGCUUUCUUGAA | Jaillon *et al.*, 2007 |
| Vv-miR396d | UUCCACAGCUUUCUUGAACUG | UUCCACAGCUUUCUUGAA | Jaillon *et al.*, 2007 |
| Vv-miR396e | UUCCACGGCUUUCUUGAACUU | UUCCACGGCUUUCUUGAACUU | Cai *et al.*, 2008 |
| Vv-miR396f | UUCCACAGCUUUCUUGAACUG | UUCCACAGCUUUCUUGAACUG | Cai *et al.*, 2008 |
| Vv-miR397a | UCAUUGAGUGCAGCGUUGAUG | CAUUGAGUGCAGCGUUGAUGA | Jaillon *et al.*, 2007/ Cai *et al.,* 2008 |
| Vv-miR397b | UCAUUGAGUGCAGCGUUGAUG | CAUUGAGUGCAGCGUUGAUGA | Jaillon *et al.*, 2007 |
| Vv-miR398a | UGUGUUCUCAGGUCACCCCUU | UGUGUUCUCAGGUCACCCCUU | Jaillon *et al.*, 2007 |
| Vv-miR398b | UGUGUUCUCAGGUCACCCCUG | UGUGUUCUCAGGUCGCCCCUG | Jaillon *et al.*, 2007/ Cai *et al.,* 2008 |
| Vv-miR398c | UGUGUUCUCAGGUCACCCCUG | UGUGUUCUCAGGUCGCCCCUG | Jaillon *et al.*, 2007Cai *et al.,* 2008 |
| Vv-miR399a | UGCCAAAGGAGAAUUGCCCUG | UGCCAAAGGAGAAUUGCC | Jaillon *et al.*, 2007 |
| Vv-miR399b | UGCCAAAGGAGAGUUGCCCU**G** | GCCAAAGGAGAGUUGCCCU | Jaillon *et al.*, 2007 |
| Vv-miR399c | UGCCAAAGGAGAGUUGCCCU**G** | GCCAAAGGAGAGUUGCCCU | Jaillon *et al.*, 2007 |
| Vv-miR399d | UGCCAAAGGAGAUUUGCUC**GU** | UCUGCCAAAGGAGAUUUGCUC | Jaillon *et al.*, 2007 |
| Vv-miR399e | UGCCAAAGGAGAUUUGCCCGG | UGCCAAAGGAGAUUUGCCCGG | Jaillon *et al.*, 2007 |
| Vv-miR399g | UGCCAAAGGAGAUUUGCCCCU | UGCCAAAGGAGAUUUGCCCCU | Jaillon *et al.*, 2007 |
| Vv-miR399h | UGCCAAAGGAGAAUUGCCCUG | UGCCAAAGGAGAAUUGCCC | Jaillon *et al.*, 2007 |
| Vv-miR399i | CGCCAAAGGAGAGUUGCCCUG | CGCCAAAGGAGAGUUGCCC | Jaillon *et al.*, 2007 |
| Vv-miR399i | UGCCAAAGGAGAGUUGCCCUG | CGCCAAAGGAGAGUUGCCC | Cai *et al.,* 2008 |
| Vv-miR403a | UUAGAUUCACGCACAAACUCG | UUAGAUUCACGCACAAACU | Jaillon *et al.*, 2007/ Cai *et al.,* 2008 |
| Vv-miR403b | UUAGAUUCACGCACAAACUCG | UUAGAUUCACGCACAAACU | Jaillon *et al* , 2007/ Cai *et al.,* 2008 |
| Vv-miR403c | UUAGAUUCACGCACAAACUCG | UUAGAUUCACGCACAAACU | Jaillon *et al.*, 2007/Cai *et al.,* 2008 |
| Vv-miR403d | UUAGAUUCACGCACAAACUCG | UUAGAUUCACGCACAAACU | Jaillon *et al.*, 2007 |
| Vv-miR403e | UUAGAUUCACGCACAAACUCG | UUAGAUUCACGCACAAACU | Jaillon *et al.*, 2007 |
| Vv-miR403f | UUAGAUUCACGCACAAACUCG | UUAGAUUCACGCACAAACU | Jaillon *et al.*, 2007 |
| Vv-miR408 | AUGCACUGCCUCUUCCCUGGC | AUGCACUGCCUCUUCCCUGGC | Jaillon *et al.*, 2007 |
| Vv-miR477(477a) | AUCUCCCUCAAAGGCUUCCAA | CUCCCUCAAAGGCUUCCA | Jaillon *et al.*, 2007 |
| Vv-miR477a | CUCCCUCAAAGGCUUCCA | CUCCCUCAAAGGCUUCCA | Cai *et al.,* 2008 |
| Vv-miR477b | CUCCCUCAAAGGCUUCCA | CUCCCUCAAAGGCUUCCA | Cai *et al.*, 2008 |
| Vv-miR477c | CUCCCUCAAAGGCUUCCA | CUCCCUCAAAGGCUUCCA | Cai *et al.*, 2008 |
| Vv-miR477d | UCCCUCAAAGGCUUCCAA | CUCCCUCAAAGGCUUCCA | Cai *et al.*, 2008 |
| Vv-miR477f | CUCCCUCAAAGGCUUCCA | CUCCCUCAAAGGCUUCCA | Cai *et al.*, 2008 |
| Vv-miR477g | CUCCCUCAAAGGCUUCCA | CUCCCUCAAAGGCUUCCA | Cai *et al.*, 2008 |
| Vv-miR477-3p | GUUGGAGGCCUUCGUGGGACA | GUUGGAGGCCUUCGUGGGACA | Cai *et al.*, 2008 |
| Vv-miR479 | UGUGGUAUUGGUUCGGCUCAUC | UGUGGUAUUGGUUCGGCUCAUC | Jaillon *et al.*, 2007 |
| Vv-miR482 | CCUACUCCUCCCAUUCC | UCUUUCCUACUCCUCCCAUUCC | Jaillon *et al.*, 2007 |
| Vv-miR482 | UCUUUCCUACUCCUCCCAUUCC | UCUUUCCUACUCCUCCCAUUCC | Cai *et al.,* 2008 |
| Vv-miR529 | AGAAGAGAGAGAGUACAGCU | AGAAGAGAGAGAGUACAGCU | Cai *et al.*, 2008 |
| Vv-miR535a | UGACAACGAGAGAGAGCACGCU | UGACAACGAGAGAGAGCACGC | Jaillon *et al.*, 2007 |
| Vv-miR535b | UGACAACGAGAGAGAGCACGCU | UGACAACGAGAGAGAGCACGC | Jaillon *et al.*, 2007 |
| Vv-miR535c | UGACAACGAGAGAGAGCACGCU | UGACAACGAGAGAGAGCACGC | Jaillon *et al.*, 2007 |
| Vv-miR535d | UGACAACGAGAGAGAGCACGCU | UGACAACGAGAGAGAGCACGC | Jaillon *et al.*, 2007 |
| Vv-miR535e | UGACAACGAGAGAGAGCACGCU | UGACAACGAGAGAGAGCACGC | Jaillon *et al.*, 2007 |
| Vv-miR535f | UGACAACGAGAGAGAGCAUGC | UGACAACGAGAGAGAGCAUGC | Cai *et al.*, 2008 |
| Vv-miR535g | UGACAAAGAGAGAGAGCACAC | UGACAAAGAGAGAGAGCACAC | Cai *et al.*, 2008 |
| Vv-miR535h | UGACAAAGAGAGAGAGCACAC | UGACAAAGAGAGAGAGCACAC | Cai *et al.*, 2008 |
| Vv-miR535i | UGACAGCGAGAGAGAGCACAC | UGACAGCGAGAGAGAGCACAC | Cai *et al.*, 2008 |
| Vv-miR827 | UUAGAUGAUCAUCAACAAAC | UUAGAUGAUCAUCAACAAAC | Cai *et al.*, 2008 |
| Vv-miR828a | UCUUGCUCAAAUGAGCAUUCCA | UCUUGCUCAAAUGAGCAUUCCA | Jaillon *et al.*, 2007/ Cai *et al.,* 2008 |
| Vv-miR828b | UCUUGCUCAAAUGAGUAUUCCA | UCUUGCUCAAAUGAGUAUUCCA | Jaillon *et al.*, 2007/ Cai *et al.,* 2008 |
| Vv-miR845a | UGCAUGCUCUGAUACCAAUUGAUG | AGCUCUGAUACCAAUUGAUAAAAC | Cai *et al.,* 2008 |
| Vv-miR845a | UAGCUCUGAUACCAAUUGAUA | AGCUCUGAUACCAAUUGAUAAAAC | Jaillon *et al.*, 2007 |
| Vv-miR845b | UAGCUCUGAUACCAAUUGAUA | AGCUCUGAUACCAAUUGAUAAAAC | Jaillon *et al.*, 2007 |
| Vv-miR845b | GCAUGCUCUGAUACCAAUUGAUG | AGCUCUGAUACCAAUUGAUAAAAC | Cai *et al.,* 2008 |
| Vv-miR845c (845-3p) | AGGCUCUGAUACCAAUUGAUG | AGGCUCUGAUACCAAUUGAUG | Jaillon *et al.*, 2007/ Cai *et al.,* 2008 |
| Vv-miR1030a | UCUGCAUUUGCACCUGCACCU | UCUGCAUUUGCACCUGCACCU | Cai *et al.*, 2008 |
| Vv-miR1030b | UCUGCAUUUGCACCUGCACCU | UCUGCAUUUGCACCUGCACCU | Cai *et al.*, 2008 |

Notes: Frame denotes the miRNAs having same sequences derived from different precursors; the shading regions are the same miRNAs that were predicated differently in sequences from the two reports, and all these sequences were experimentally confirmed in this study; the red bases are the different ones between the predicated sequences and validated ones, while the red ^ denotes the missing biases.
