# Supplementary material for: Characterization of microRNAs Identified in a Table Grapevine Cultivar with Validation of Computationally Predicted Grapevine miRNAs by miR-RACE
Source: PLoS One. 2011 Jul 28;6(7):e21259. doi: 10.1371/journal.pone.0021259 (PMC3145640; doi:10.1371/journal.pone.0021259)
Supplement: Table S2 — Predicted target genes or proteins for grapevine miRNAs validated by miR-RACE. (DOC) [file pone.0021259.s003.doc]

| **Table S2** | | |  |
| --- | --- | --- | --- |
| **Grapevine miRNA family** | **Identified grapevine miRNAs** | **Predicted target genes or proteins** | **Annotated functions** |
| Vv-miR156 | Vv-miR156a, b, c, d, e, f, g, h, i | Squamosa-promoter binding protein (SPB), SPB1, SPB2, SPB3, SPB4; Squamosa promoter-binding protein-like1(SPL1), SPL2, SPL3, SPL4, SPL5, SPL6, SPL9, SPL10, SPL11, SPL12; Phox/Bem1p (PB1) | Transcription factor activity, DNA binding; Unknown |
| Vv-miR159 | Vv-miR159a, b, c | MYB007,MYB124 | Transcription factor |
| Vv-miR160 | Vv-miR160a, b, c, d, e, f | Auxin response factor 3 (ARF3), ARF10, ARF16; Cell wall protein 3 (CWP3), zinc finger protein | Auxin-responsive activity, signal transduction; Drought stress-related |
| Vv-miR162 | Vv-miR162 | No target gene |  |
| Vv-miR164 | Vv-miR164a, b, c, d | NAC domain protein (NAC5, NAC15, NAC21, NAC28, NAC80, NAC100); No apical meristem (NAM) –like protein, nam-like protein 18 (NH18) , NH14, NAM / CUC2, NAC2, NAC6, NAC17, NAC19 | Transcription factor activity |
| Vv-miR166 | Vv-miR166a, b, c, d, e, f, g, h | No target gene |  |
| Vv-miR167 | Vv-miR167a, b, c, d, e | ARF3, ARF4, ARF6, ARF8; RAD51-like 1 (S. *cerevisiae*) protein | Auxin-responsive activity, signal transduction; Unknown |
| Vv-miR168 | Vv-miR168 | No target gene found |  |
| Vv-miR169 | Vv-miR169a, b, c, d, e, f, j, h, I, j, k, l, m, n, o, p, q, r, s, t, u, v, w, x | Nuclear transcription factor Y subunit A-1 (NF-YA1), NF-YA3,NF-YA8 | Nuclear transcription factor activity |
| Vv-miR171 | Vv-miR171a, b, c, d, e, f, h, i | GRAS family transcription factor (GRAS58-64), scarecrow-like transcription factor 6 (SCL6), Scl1 protein;SCL6-IV (SCL6-IV), GAI-like protein 1 (GAI1), Scl1 protein, GRAS transcription factor family protein; SCL-III, SCL-IV | Transcription factor activity |
| Vv-miR172 | Vv-miR172c, d | AP2, TOE domain-containing transcription factor | Transcription factor activity |
| Vv-miR319 | Vv-miR319b, c, e, f, g | MYB007, MYB012, MYB157, r2r3-MYB, MYB81, MYB124; Peptidylprolyl isomerase A (cyclophilin A) | Transcription factor activity; enzyme activity |
| Vv-miR390 | Vv-miR390 | TAS3a1, TAS3-like trans-acting siRNA gene; At1g73070/F3N23_27 | Unknown |
| Vv-miR393 | Vv-miR393a, b | F-box family protein (FBL3), FBL4; auxin signaling F-box1 (AFB1), AFB2, AFB3; Transport inhibitor response protein (TIR1) | Signal-transduction; Transport inhibitor activity |
| Vv-miR394 | Vv-miR394a, b, c | F-box family protein | Transcription factor |
| Vv-miR395 | Vv-miR395a, b, c, d, e, f, g, h, I, j, k, l, m | ATP sulfurylase 1(APS1), APS2, APS3, APS4, ATP sulfurylase/APS kinase | ATP sulfurylase activity |
| Vv-miR396 | Vv-miR396a, b, c, d | No target gene found |  |
| Vv-miR397 | Vv-miR397a, b | laccase-like1(lac1), lac2, lac3, lac4, lac5, lac6, lac11, lac17, lac110; AT-rich element binding factor 2 (ATF2); MLO-like protein 6 | Laccase activity; Unknown |
| Vv-miR398 | Vv-miR398a, b, c | No target gene found |  |
| Vv-miR399 | Vv-miR399a, b, c, d, e, g, h, i | Resveratrol synthase ( labst1 ), stilbene synthase (STS), stilbene synthase 1 (St1), vinst1, resveratrol synthase |  |
| Vv-miR403 | Vv-miR403a, b, c, d, e, f | Translation initiation factor 3;unknown protein (AT1G72480) | Translation initiation activity; Unknown |
| Vv-miR408 | Vv-miR408 | Copper ion binding ; Copper ion binding / electron carrier (ARPN); Calcium binding protein (CBP gene); putative basic blue protein (plantacyanin) | Encodes plantacyanin; electron carrier activity, copper ion binding |
| Vv-miR477 | Vv-miR477 | F-box family protein, expressed protein, hypothetical protein | Unknown |
| Vv-miR479 | Vv-miR479 | No target gene found |  |
| Vv-miR482 | Vv-miR482 | Disease resistance gene analog NBS-LRR-like protein (ARGA-1), NBS-LRR resistance-like protein RGC754; Zinc finger (C2H2 type) family protein, NBS-LRR class resistance protein (BR-9), putative hexose transporter (HT6); SNAP receptor/ protein transporter (BS14A), Betip/sftip-like protein 14A (BS14A) | Disease resistance activity; ranscription factor activity, zinc ion binding, nucleic acid binding; disease resistance activity; ripening related expression parallel to the rate of hexose storage; Protein transporter activity, SNAP receptor activity |
| Vvi-miR529 | Vvi-miR529 | GTP binding protein, Sar1 homolog (SAR1), SAR1/GTP-binding secretory factor, Secretion-associated RAS super family 2 ( SAR2), SAR1-like GTP binding protein; SPL1, SPL5, SPL9 | Transcription factor; GTPase activity; GTP binding |
| Vv-miR535 | Vv-miR535a, b, c, d, e, i | Auxin efflux carrier family protein (AEF); Delta-N p63/p73-like protein, light-harvesting complex I protein Lhca1 (Lhca1-2); predicted protein (PHYPADRAFT_75984); top1 mRNA for topoisomerase I | Hydrogen symporter activity; Unknown |
| Vv-miR827 | Vv-miR827 | Nitrogen limitation adaptation(NLA); ubiquitin -protein ligase (NLA); putative TNP2-like protein and putative TNP1-like protein; MYB156, MYB221, MYB4a, MYB4b | Encodes a likely ubiquitin E3 ligase with RING and SPX domains that is involved in mediating immune responses; Transcription factor; putative negative regulation of transcription |
| Vv-miR828 | Vv-miR828a, b | MYB0, MYB1, MYB2, MYB 3, MYB4, MYB5, MYB113, MYB129, MYB165, MYB171, MYB194, MYB205; MYB66, MYB071, MYB072, MYB109, MYB143, glabrous 1 (GL1, MYB0), werewolf (WER); ATP synthase epsilon subunit (atp E) | Encodes a MyB-related protein, transcription factor activity; RING and SPX domains that is involved in mediating immune responses; transcription factor activity |
| Vv-miR845 | Vv-miR845a, b | No target gene found |  |
| Vvi-miR1030 | Vvi-miR1030 | Gamma vacuolar precessing enzyme (Gamma-VPE), Cysteine-type endopeptidase (Gamma-VPE), Legumain-like protease (see2b gene), Legumain-like protease (see2a gene), vacuolar processing enzyme (Gamma-VPE) | Vacuolar processing enzyme activity |
