# Supplementary material for: Characterization of microRNAs Identified in a Table Grapevine Cultivar with Validation of Computationally Predicted Grapevine miRNAs by miR-RACE
Source: PLoS One. 2011 Jul 28;6(7):e21259. doi: 10.1371/journal.pone.0021259 (PMC3145640; doi:10.1371/journal.pone.0021259)
Supplement: Table S3 — Comparison of target genes of experimentally verified orthologous miRANs in grapevine and Arabidopsis . (DOC) [file pone.0021259.s004.doc]

| **Table S3** | | | |
| --- | --- | --- | --- |
| **Grapevine miRNA** | **Base variation compared with *Arabidopsis* miRNAs (varied base number)** | **Grapevine miRNA’s target genes [mismatch base number]** | ***Arabidopsis* miRNA’s target genes [mismatch base number]** |
| Vv-miR156a | UUGACAGAAGAGAGGGAGCAC (2) | SQUAMOSA PROMOTER-BINDING PROTEIN-LIKE 1 (SPL1), SPL9 [1]; SPL1, 3, 4, 5, SPB 0, 1, 2, 3, 4 [2]; Phox/Bem1p (PB1) domain-containing protein [4]; SPL2, 10, 11 [1]; SPL1, 4, 5, 9 (DT024646.1) [3] | SPB, SPB1, 2, 6; SPL2, 11, 13 [1],  SPB, SPL2, 13 [1]; SPL3 [2] |
| Vv-miR156b, c, d | UGACAGAAGAGAGUGAGCAC (0) | SPL1, SPL9 [0]; SPB0, SPB2, SPL1, SPL3, SPL4, SPL5, SPL9, SPL12 [2]; SPB, SPL, SPL1, SPL3, SPL4, SPL5, SPL9, SPL12 [2] | SPB, SPB1, SPL2, SPL11, SPL13 [1]; SPL3 [2]; SPL3, SPL4, SPL5 [3] |
| Vv-miR156e | UGACAGA**G**GAGAGUGAGCAC (1) | SQUAMOSA PROMOTER-BINDING PROTEIN-LIKE 5 (SPL5) [2]; UBIQUITIN CARRIER PROTEIN 7 (UBC7), UBC13, UBC14 [4] | SPL2, SPL10, SPL11 [1] |
| Vv-miR156f | **U**UGACAGAAGA**U**AGAGAGCAC (3) | SPL1, SPL2, SPL9, SPL10, SPL11 [1] | SQUAMOSA PROMOTER-BINDING PROTEIN SPB, SPB2, SPL11 [1] |
| Vv-miR156g | UUGACAGAAGAUAGAGAGCAC (4) | SPL1, SPL2, SPL9, SPL10, SPL11 [1] | SPL9; STEROL METHYLTRANSFERASE 2 (SMT2 ), SMT3 [4] |
| Vv-miR159a | ^UUGGA**G**UGAAGGGAGCUCU**C** (3) | MYB DOMAIN PROTEIN 124 (MYB124), MYB012 , MYB007, MYB157 [2] | MYB33, MYB65, MYB124, MYB157, MYB007, R2-R3 MYB [3] |
| Vv-miR159b | ^UUGGA**G**UGAAGGGAGCUCU**C** (3) | MYB124, MYB012, MYB007, MYB157 [2] | MYB33, MYB65, MYB124, MYB157, MYB007 [3] |
| Vv-miR159c | UUUGGAUUGAAGGGAGCUCUA (2) | MYB124, MYB007, MYB 012, MYB157 [4] | MYB33, MYB65, MYB124, MYB157, MYB007 [3] |
| Vv-miR160a, b | UGCCUGGCUCCCUGAAUGCCA (1) | Auxin response factor 10 (ARF10), ARF16, ARF3 [2]; cell wall protein 3 (cwp3 gene) [0] | ARF10, ARF16, ARF3 [4] |
| Vv-miR160c | UGCCUGGCUCCCUGUAUGCCA (0) | ARF2, ARF3, ARF6, ARF8, ARF10, ARF15, ARF16, ARF19, ARF21 [1] | ARF3, ARF10, ARF16 [4] |
| Vv-miR162 | UCGAUAAACCUCUGCAUCCAG (0) | No target gene found | No target gene found |
| Vv-miR164a | UGGAGAAGCAGGGCACGUGCA (0) | NAC4, NAC5, NAC15, NAC21/22, NAC28, NAC79, NAC80 [2]; NAC4,NAC15, NAC21/22, NAC28, NAC17, NAC19, NAM (no apical meriste)-like protein, NAM(2-1), NAM(2-2) [3] | NAC100, NAC80, NAM (no apical meriste)-mlike protein (MFB13.22) [3]; NAC1, NAC2, NAC3, NAC4, NAC5, NAC6, NAC7, NAC011, NAC34, NAC35, NAC059, NAC081, NAC087, NAC092, CUP SHAPED COTYLEDON 3 (CUC3), NAM/ CUC2 [2]; Heat shock protein (HSP17.6 , HSP17.8) [4] |
| Vv-miR164b | UGGAGAAGCAGGGCACAUGCU (2) | NAC DOMAIN CONTAINING PROTEIN ,TRANSCRIPTION FACTOR 1 (NAC1), NAC3, NAC028, NAC070, NAC081, CUP-SHAPED COTYLEDON 1 (CUC1), CUC2, CUC3, CUC5 [2] | NAC100, NAC080; no apical meristem-like protein (NAM/MAF13) [2] |
| Vv-miR164c | UGGAGAAGCAGGGCACGUGCA (1) | NAC5, NAC6, NAC021, NAC022, NAC080, NAC3, NAC7, NAC32, NAC015, NAC023, NAC28, NAC18, NAM/CUC2 [2] | NAC100, NAC080; NAC5, NAC028, NAC023; NAM (MFB13.22) [3] |
| Vv-miR166a | UCUCGGACCAGGCUUCAUUCC^^ (4) | No target gene found | HD-ZIP protein family (HB-15), HD-ZIP1 (HB-1), HB2, HB3, HB4, HB5, HB6, HB7, HB8, HB9 [3] |
| Vv-miR166b | UCGGACCAGGCUUCAUUCCUC (1) | No target gene found | HB-15, HB-1, HB2, HB3, HB5, HB6, HB7, HB8, HB9 [3] |
| Vv-miR166c, e, d, f, g | UCGGACCAGGCUUCAUUCCCCC (0) | No target gene found | HB-15, HB-1, HB2, HB3, HB5, HB6, HB7, HB8, HB9 [3] |
| Vv-miR167a | UGAAGCUGCCAGCAUGAUCUG (1) | AUXIN RESPONSE FACTOR 6 (ARF6), ARF8 [3] | ARF6, ARF8; ARF3, ARF10, ARF16 [4] |
| Vv-miR167b | UGAAGCUGCCAGCAUGAUCUAA (1) | No target gene found | Auxin response factor 6 (ARF6), ARF8 |
| Vv-miR167c | UGAAGCUGCCAGCAUGAUCUC (3) | ARF3, ARF4, ARF6, ARF8 [4] | ARF6, ARF8 [4] |
| Vv-miR167d | UGAAGCUGCCAGCAUGAUCUA^ (2) | ARF3, ARF4, ARF6, ARF8 [4] | No target gene found |
| Vv-miR168 | UCGCUUGGUGCAGGUCGGGAA (0) | No target gene found | No target gene found |
| Vv-miR169a | CAGCCAAGGAUGACUUGCCGG (1) | Nuclear transcription factor Y subunit A-3 (NF-YA3) [4] | No target gene found |
| Vv-miR169c | CAGCCAAGGAUGACUUGCCGG (0) | NF-YA3 [4] | No target gene found |
| Vv-miR169d | CAGCCAAGGAUGACUUGCCGG (1) | NF-YA3 [4] | No target gene found |
| Vv-miR169j, k | CAGCCAAGGAUGACUUGCCGG (1) | NF-YA3 [4] | NF-YA3 [3]; 窗体顶端  CHY1 (BETA- HYDROXYISOBUTYRYL- COA HYDROLASE 1); 3-hydroxyisobutyryl-CoA hydrolase (CHY1) [3]窗体底端 |
| Vv-miR169m | UGAGCCAAGGAUGACUUGCCG**^^**(4) | NF-YA3 [4] | NF-YA3 [3]; CHY1 (BETA- HYDROXYISOBUTYRYL- COA HYDROLASE 1); 3-hydroxyisobutyryl-CoA hydrolase (CHY1) [3] |
| Vv-miR169b | **U**GAGCCAAGGAUGGCUUGCCGU (3) | No target gene found | No target gene found |
| Vv-miR169h | **U**GAGCCAAGGAUGGCUUGCCGU (4) | No target gene found | No target gene found |
| Vv-miR169e | U^AGCCAAGGAUGACUUGCCUGC (4) | No target gene found | No target gene found |
| Vv-miR169f, g | C^AGCCAAGGAUGACUUGCCGA (4) | NF-YA3 [4] | No target gene found |
| Vv-miR169i | **U**GAGCCAAGGAUGACUGGCCGU (5) | No target gene found | 窗体顶端  CHY1 (BETA- HYDROXYISOBUTYRYL- COA HYDROLASE 1); 3-hydroxyisobutyryl- CoA hydrolase (CHY1) [3]窗体底端 |
| Vv-miR169l | UGAGCCAAGGAUGACUUGCC^G (3) | NF-YA3 [4] | NF-YA3 [3]; CHY1 [3] |
| Vv-miR169n | UAGAGCCAAGGAUGACUUGCC^G (3) | No target gene found | NF-YA3 [3]; CHY1 [3] |
| Vv-miR171c | UGAUUGAGCCGUGCCAAUAUC^^^ | GRAS family (GRAS58-65); SCL6, SCL6-IV[1] | GRAS58-63, SCL6, SCL6-III, SCL6-IV [4] |
| Vv-miR172c | GGAAUCUUGAUGAUGCUGCAG (1) | APETALA2 (AP2) [1] | AP2 [1] |
| Vv-miR172d | AGAAUCUUGAUGAUGCUGCAU (1) | AP2 [2] | AP2 [1] |
| Vv-miR172e | GGAAUCUUGAUGAUGCUGCAU (0) | AP2, TOE1[2]; TOE2 [3]; GTP-binding protein ATGB1 [4] | TOE2 [2]; AP2, TOE3 [3] |
| Vv-miR390 | AAGCUCAGGAGGGAUAGCGCC (0) | No target gene found | No target gene found |
| Vv-miR319b | UUGGACUGAAGGGAGCUCCC^ (1) | MYB DOMAIN PROTEIN 007 (MYB007), MYB012, MYB81, MYB124, MYB157 [3] | MYB33, MYB65, MYB124, MYB157, MYB007, R2-R3 MYB [3] |
| Vv-miR319c | UUGGACUGAAGGGAGCUCCC^ (2) | MYB DOMAIN PROTEIN 007 (MYB007), MYB012, MYB81, MYB124, MYB157 [3] | MYB33, MYB65, MYB124, MYB157, MYB007, R2-R3 MYB [3] |
| Vv-miR393a | UUCCAAAGGGAUCGCAUUGAU^^ (1) | Auxin signaling F-box 3 (AFB3), AFB4, AFB2, f-box family protein (FBL3), FBL2, FBL4, transport inhibitor response protein (TIR1), auxin-responsive factor TIR1-like protein (TIR1) [3] | AFB3, AFB2, FBL4, FBL2, FBL3, TIR1 [4] |
| Vv-miR393b | UCCAAAGGGAUCGCAUUGAU^^ (2) | FBL3, FBL4, AFB2, AFB3, TIR1 [2] | AFB3, AFB2, FBL4, FBL2, FBL3, TIR1 [4] |
| Vv-miR394a, b | UUGGCAUUCUGUCCACCUCC (0) | f-box family protein [1] | F-box family protein [1] |
| Vv-miR395a, d, e | CUGAAGUGUUUGGGGGAACUC (0) | ATP sulfurylase (APS1); APS2, APS4 [3] | APS1, APS2, APS3, APS4 [2]; sulfate transmembrane transporter (AST68), sulfate transporter protein [3]; APS1, APS4 [4] |
| Vv-miR395b, c, f | CUGAAGUGUUUGGGGGAACUC (1) | ATP sulfurylase 1 (APS1), APS2, APS3, APS4 [3] | No target gene found |
| Vv-miR396a | UUCCACAGCUUUCUUGAA^^^ (3) | No target gene found | No target gene found |
| Vv-miR397a | ^CAUUGAGUGCAGCGUUGAUGA (2) | AT-rich element binding factor 2 (ATF2); MLO-like protein 6 (MLO6) [0]; Laccase 2-1 (Lac2-1), Lac2-2, Lac2-3, lac1,2, 3, 4, 5, 6, 110b, 110c [2] | CASEIN KINASE II BETA CHAIN 1 (CKB1), CKB2, CKB3, CKB4 [4]; lac1, 2, 3, 4, 5, 6, 110b, 110c, Lac2-1, Lac2-2, Lac2-3 [3] |
| Vv-miR397b | ^CAUUGAGUGCAGCGUUGAUGA (3) | AT-rich element binding factor 2 (ATF2); MLO-like protein 6 (MLO6) [0]; Laccase 2-1 (Lac2-1), Lac2-2, Lac2-3, lac1, 2, 3, 4, 5, 6, 110b,110c [2] | CASEIN KINASE II BETA CHAIN 1 (CKB1), CKB2, CKB3, CKB4 [3] |
| Vv-miR398a | UGUGUUCUCAGGUCACCCCUU (0) | No target gene found | No target gene found |
| miR398b, c | UGUGUUCUCAGGUCGCCCCUG (1) | No target gene found | No target gene found |
| miR399a | UGCCAAAGGAGAAUUGCCCUG (1) | No target gene found | PHOSPHATE 2 (PHO2) [2] |
| miR399b, c | UGCCAAAGGAGAGUUGCCCU^ (1) | No target gene found | PHO2; E2, ubiquitin-conjugating enzyme [3] |
| miR399d | UGCCAAAGGAGAUUUGCUC^^ (3) | Acid phosphatase type 5 (acp5) [4] | PHO2; E2, ubiquitin-conjugating enzyme [2] |
| miR399e | UGCCAAAGGAGAUUUGCCCGG (2) | No target gene found | PHO2 [3] |
| Vv-miR403a, b, c | UUAGAUUCACGCACAAACU^^ (2) | No target gene found | No target gene found |
| Vv-miR408 | AUGCACUGCCUCUUCCCUGGC (0) | ARPN (PLANTACYANIN), APRN(copper ion binding/ electron carrier) [4] (EC939917.1); calcium binding protein (CBP gene), calmodulin-related protein (PPRG1) | ARPN (PLANTACYANIN), copper ion binding/ electron carrier (ARPN) [3], basic blue protein (plantacyanin) |
| miR827 | UUAGAUGAUCAUCAACAAAC^ (2) | Nitrogen limitation adaptation (NLA) [3] | NLA [1] |
| miR828a | UCUUGCUCAAAUGAGUAUUCCA (1) | AtpE gene, tRNA-Val, tRNA-Met gene [1] | No target gene found |

Notes: The red bases is the various bases of Vv-miRANs sequences compared with their orthologous ones in *Arabidopsis*; the red ^ denotes the missing bases in Vv-miRNAs sequeces compared with their orthologous one in *Arabidopsis.*
