# Supplementary material for: Characterization of microRNAs Identified in a Table Grapevine Cultivar with Validation of Computationally Predicted Grapevine miRNAs by miR-RACE
Source: PLoS One. 2011 Jul 28;6(7):e21259. doi: 10.1371/journal.pone.0021259 (PMC3145640; doi:10.1371/journal.pone.0021259)
Supplement: Table S4 — Primers used for miR-5′RACE, miR-3′RACE. (DOC) [file pone.0021259.s005.doc]

| **Table S4** | | |
| --- | --- | --- |
| **Vv-miRNAs** | **MSP1 (5’→3’)** | **MSP2 (5’→3’)** |
| Vv-miR156a | TTTTTTTTTTGTGCTCCCTCTCTTCTG | GGAGTAGAAATGACAGAAGAGAGGGA |
| Vv-miR156b, c, d | TTTTTTTTTTGTGCTCACTCTCTTCTG | GGAGTAGAAATGACAGAAGAGAGTGAG |
| Vv-miR156e | TTTTTTTTTTGTGCTCACTCTCCTCTG | GGAGTAGAAATGACAGAGGAGAGTGAG |
| Vv-miR156f, g | TTTTTTTTTTGTGCTCTCTATCTTCTG | GGAGTAGAAATTGACAGAAGATAGAGA |
| Vv-miR156i | TTTTTTTTTTGTGCTCTCTATCTTCTG | GGAGTAGAAATGACAGAAGATAGAGAG |
| Vv-miR156h | TTTTTTTTTTATGCTCTCTCTCTTCTG | GGAGTAGAAATGACAGAAGAGAGTGAG |
| Vv-miR159a, b | TTTTTTTTTTGAGAGCTCCCTTCACTC | GGAGTAGAAACTTGGAGTGAAGGGAGC |
| Vv-miR159c | TTTTTTTTTTTAGAGCTCCCTTCAATC | GGAGTAGAAATTTGGATTGAAGGGAGC |
| Vv-miR159d | TTTTTTTTTTTAGGAGCTCCCTTCAGTC | GGAGTAGAAATTTGGACTGAAGGGAGC |
| Vv-miR159e | TTTTTTTTTTTAGAGCTCCCTTCAGTC | GGAGTAGAAAATTGGACTGAAGGGAGC |
| Vv-miR160a, b, e | TTTTTTTTTTTGGCATTCAGGGAGCCA | GGAGTAGAAATGCCTGGCTCCCTGAAT |
| Vv-miR160c, d, f | TTTTTTTTTTTGGCATACAGGGAGCCA | GGAGTAGAAATGCCTGGCTCCCTGTAT |
| Vv-miR162 | TTTTTTTTTTCTGGATGCAGAGGTTTA | GGAGTAGAAATCGATAAACCTCTGCAT |
| Vv-miR164a, c, d | TTTTTTTTTTTGCACGTGCCCTGCTTC | GGAGTAGAAATGGAGAAGCAGGGCACG |
| Vv-miR164b | TTTTTTTTTTAGCATGTGCCCTGCTTC | GGAGTAGAAATGGAGAAGCAGGGCACA |
| Vv-miR166a | TTTTTTTTTTCAGGAATGAAGCCTGGT | GGAGTAGAAATCGGACCAGGCTTCATT |
| Vv-miR166b | TTTTTTTTTTGAGGAATGAAGCCTGGT | GGAGTAGAAATCGGACCAGGCTTCATT |
| Vv-miR166c, e, h | TTTTTTTTTTGGGGGAATGAAGCCTGG | GGAGTAGAAATCGGACCAGGCTTCATT |
| Vv-miR166d, g, f | TTTTTTTTTTAGGGGAATGAAGCCTGG | GGAGTAGAAATCGGACCAGGCTTCATT |
| Vv-miR167a | TTTTTTTTTTCAGATCATGCTGGCAGC | GGAGTAGAAATGAAGCTGCCAGCATGA |
| Vv-miR167b, d, e | TTTTTTTTTTTAGATCATGCTGGCAGC | GGAGTAGAAATGAAGCTGCCAGCATGA |
| Vv-miR167c | TTTTTTTTTTTGAGATCATGCTGGCAGC | GGAGTAGAAATGAAGCTGCCAGCATGA |
| Vv-miR168 | TTTTTTTTTTTTCCCGACCTGCACCAA | GGAGTAGAAATCGCTTGGTGCAGGTCG |
| Vv-miR169a, c, d, j, k, m, p, s | TTTTTTTTTTCCGGCAAGTCATCCTTG | GGAGTAGAAACAGCCAAGGATGACTTG |
| Vv-miR169b | TTTTTTTTTTCGGCAAGCCATCCTTGG | GGAGTAGAAATGAGCCAAGGATGGCTT |
| Vv-miR169b* | TTTTTTTTTTCCGGCAATTCATCCTTG | GGAGTAGAAAAAGCCAAGGATGAATTG |
| Vv-miR169h | TTTTTTTTTTCGGCAAGCCATCCTTGG | GGAGTAGAAATGAGCCAAGGATGGCTT |
| Vv-miR169h* | TTTTTTTTTTACGGCAAGCCATCCTTG | GGAGTAGAAAGAGCCAAGGATGGCTTG |
| Vv-miR169e | TTTTTTTTTTCAGGCAAGTCATCCTTG | GGAGTAGAAATAGCCAAGGATGACTTG |
| Vv-miR169f | TTTTTTTTTTTCGGCAAGTCATCCTTG | GGAGTAGAAACAGCCAAGGATGACTTG |
| Vv-miR169i | TTTTTTTTTTTACGGCCAGTCATCCTT | GGAGTAGAAAGAGCCAAGGATGACTGG |
| Vv-miR169l | TTTTTTTTTTTACGGCAAGTCATCCTT | GGAGTAGAAAGAGCCAAGGATGACTTG |
| Vv-miR169w | TTTTTTTTTTCCGGCAAGTCATCCTTG | GGAGTAGAAACAGCCAAGGATGACTTG |
| Vv-miR169w* | TTTTTTTTTTCCGGCAAGTCATCCTTG | GGAGTAGAAAGAGCCAAGGATGACTTG |
| Vv-miR169n | TTTTTTTTTTTCCGGCAAGTCATCCTT | GGAGTAGAAAGAGCCAAGGATGACTTG |
| Vv-miR169q | TTTTTTTTTTTCCGGCAAGTCATCCTT | GGAGTAGAAAGAGCCAAGGATGACTTG |
| Vv-miR169q* | TTTTTTTTTTCGGCAAGCCATCCTTGG | GGAGTAGAAATGAGCCAAGGATGGCTT |
| Vv-miR169o | TTTTTTTTTTTGCGGCAAGTCATCCTT | GGAGTAGAAAGAGCCAAGGATGACTTG |
| Vv-miR169o* | TTTTTTTTTTTCGGCAAGTCATCCTTG | GGAGTAGAAATGAGCCAAGGATGACTT |
| Vv-miR169x | TTTTTTTTTTTTAGGCAAGTCATCCTT | GGAGTAGAAATAGCCAAGGATGACTTG |
| *Vv-miR169y | TTTTTTTTTTTAGGCAAGTCATCCTTC | GGAGTAGAAATAGCGAAGGATGACTTG |
| Vv-miR169r, u | TTTTTTTTTTCGGCAAGTCATCCTTGA | GGAGTAGAAATGAGTCAAGGATGACTT |
| Vv-miR169t | TTTTTTTTTTCGGCAAGTCATCCTTGA | GGAGTAGAAACGAGTCAAGGATGACTT |
| Vv-miR169v | TTTTTTTTTTCCGGCAATTCATCCTTG | GGAGTAGAAAAAGCCAAGGATGAATTG |
| Vv-miR169v* | TTTTTTTTTTCGGCAAGTCATCCTTGG | GGAGTAGAAATGAGCCAAGGATGACTT |
| Vv-miR171a | TTTTTTTTTTCATGATATTGGCACGGC | GGAGTAGAAATTGAGCCGTGCCAATAT |
| Vv-miR171b | TTTTTTTTTTGATATTGACGCGGCTCA | GGAGTAGAAATGATTGAGCCGCGTCAA |
| Vv-miR171c, d | TTTTTTTTTTCGTGATATTGGCACGGC | GGAGTAGAAATTGAGCCGTGCCAATAT |
| Vv-miR171e | TTTTTTTTTTGATATTGGCGCGGCTCA | GGAGTAGAAATGATTGAGCCGCGCCAA |
| Vv-miR171f | TTTTTTTTTTAGTGATATTGGCGCGGC | GGAGTAGAAATTGAGCCGCGCCAATAT |
| Vv-miR171g | TTTTTTTTTTGGTGATATTGGTTCGGC | GGAGTAGAAATTGAGCCGAACCAATAT |
| Vv-miR171h | TTTTTTTTTTGATATTGGCGCGGCTCA | GGAGTAGAAATGGTTGAGCCGCGCCAA |
| Vv-miR171i | TTTTTTTTTTGATGATATTGGCACGGC | GGAGTAGAAATTGAGCCGTGCCAATAT |
| *Vv-miR171j, k | TTTTTTTTTTGATATTGGCACGGCTCA | GGAGTAGAAATTGATTGAGCCGTGCCA |
| Vv-miR172c | TTTTTTTTTTCTGCAGCATCATCAAGA | GGAGTAGAAAGGAATCTTGATGATGCT |
| Vv-miR172d | TTTTTTTTTTATGCAGCATCATCAAGA | GGAGTAGAAAAGAATCTTGATGATGCT |
| Vv-miR172e | TTTTTTTTTT TGAATCTTGATGATGCT | GGAGTAGAAA ATGGAGCATCATCAAGA |
| Vv-miR319b, c, f | TTTTTTTTTTGGGAGCTCCCTTCAGTC | GGAGTAGAAACTTGGACTGAAGGGAGC |
| Vv-miR319e | TTTTTTTTTTAGGAGCTCCCTTCAGTC | GGAGTAGAAATTTGGACTGAAGGGAGC |
| Vv-miR319g | TTTTTTTTTTGGATCAATGCGATCCCT | GGAGTAGAAAATTGGACTGAAGGGAGC |
| Vv-miR390a, b* | TTTTTTTTTTGGCGCTATCCCTCCTGA | GGAGTAGAAAAAGCTCAGGAGGGATAG |
| Vv-miR393a, b | TTTTTTTTTTGGATCAATGCGATCCCT | GGAGTAGAAATCCAAAGGGATCGCATT |
| Vv-miR393b* | TTTTTTTTTTGGGATCAATGCGATCCC | GGAGTAGAAATCCAAAGGGATCGCATT |
| Vv-miR394a, b, c | TTTTTTTTTTGGAGGTGGACAGAATGC | GGAGTAGAAATTGGCATTCTGTCCACC |
| Vv-miR395a, b, c, d, e, f, h, I, j, k, l, m | TTTTTTTTTTGAGTTCCCCCAAACACT | GGAGTAGAAACTGAAGTGTTTGGGGGA |
| Vv-miR396a | TTTTTTTTTTTAGTTCAAGAAAGCTGT | GGAGTAGAAATTCCACAGCTTTCTTGA |
| Vv-miR396b | TTTTTTTTTTAGTTCAAGAAAGCTGTG | GGAGTAGAAATTCCACAGCTTTCTTGA |
| Vv-miR396c, d | TTTTTTTTTTCAGTTCAAGAAAGCTGT | GGAGTAGAAATTCCACAGCTTTCTTGA |
| Vv-miR396e | TTTTTTTTTTAAGTTCAAGAAAGCCGT | GGAGTAGAAATTCCACGGCTTTCTTGA |
| Vv-miR396f | TTTTTTTTTTCAGTTCAAGAAAGCTGT | GGAGTAGAAATTCCACAGCTTTCTTGA |
| Vv-miR397a, b | TTTTTTTTTTCATCAACGCTGCACTCA | GGAGTAGAAATCATTGAGTGCAGCGTT |
| Vv-miR398a | TTTTTTTTTTAAGGGGTGACCTGAGAA | GGAGTAGAAATGTGTTCTCAGGTCACC |
| Vv-miR398b, c | TTTTTTTTTTGAGGGGTGACCTGAGAA | GGAGTAGAAATGTGTTCTCAGGTCACC |
| Vv-miR399a, h | TTTTTTTTTTCAGGGCAATTCTCCTTT | GGAGTAGAAATGCCAAAGGAGAATTGC |
| Vv-miR399b, c | TTTTTTTTTTCAGGGCAACTCTCCTTT | GGAGTAGAAATGCCAAAGGAGAGTTGC |
| Vv-miR399d | TTTTTTTTTTACGAGCAAATCTCCTTT | GGAGTAGAAATGCCAAAGGAGATTTGC |
| Vv-miR399e | TTTTTTTTTTCCGGGCAAATCTCCTTT | GGAGTAGAAATGCCAAAGGAGATTTGC |
| Vv-miR399g | TTTTTTTTTTAGGGGCAAATCTCCTTT | GGAGTAGAAATGCCAAAGGAGATTTGC |
| Vv-miR399i | TTTTTTTTTTCAGGGCAACTCTCCTTT | GGAGTAGAAACGCCAAAGGAGAGTTGC |
| Vv-miR399i* | TTTTTTTTTTCAGGGCAACTCTCCTTT | GGAGTAGAAATGCCAAAGGAGAGTTGC |
| Vv-miR403a, b, c, d, e, f | TTTTTTTTTTCGAGTTTGTGCGTGAAT | GGAGTAGAAATTAGATTCACGCACAAA |
| Vv-miR408 | TTTTTTTTTTGCCAGGGAAGAGGCAGT | GGAGTAGAAAATGCACTGCCTCTTCCC |
| Vv-miR477a* | TTTTTTTTTTTGGAAGCCTTTGAGGGA | GGAGTAGAAACTCCCTCAAAGGCTTCC |
| Vv-miR477a | TTTTTTTTTTTTGGAAGCCTTTGAGGG | GGAGTAGAAAATCTCCCTCAAAGGCTT |
| Vv-miR477b*, c*, f*, g* | TTTTTTTTTTTGGAAGCCTTTGAGGGA | GGAGTAGAAACTCCCTCAAAGGCTTCC |
| Vv-miR477d* | TTTTTTTTTTTTGGAAGCCTTTGAGGG | GGAGTAGAAATCCCTCAAAGGCTTCCA |
| Vv-miR477-3p* | TTTTTTTTTTTGTCCCACGAAGGCCTC | GGAGTAGAAAGTTGGAGGCCTTCGTGG |
| Vv-miR479 | TTTTTTTTTTGATGAGCCGAACCAATA | GGAGTAGAAATGTGGTATTGGTTCGGC |
| Vv-miR482 | TTTTTTTTTTCCTACTCCTCCCATTCC | GGAGTAGAAAGGAATGGGAGGAGTAGG |
| Vv-miR482* | TTTTTTTTTTGGAATGGGAGGAGTAGG | GGAGTAGAAACTTTCCTACTCCTCCCA |
| Vv-miR535a, b, c, d, e | TTTTTTTTTTAGCGTGCTCTCTCTCGT | GGAGTAGAAATGACAACGAGAGAGAGC |
| Vv-miR535f | TTTTTTTTTTGCATGCTCTCTCTCGTT | GGAGTAGAAATGACAACGAGAGAGAGC |
| Vv-miR535g, h | TTTTTTTTTTGTGTGCTCTCTCTCTTT | GGAGTAGAAATGACAAAGAGAGAGAGC |
| Vv-miR535i | TTTTTTTTTTGTGTGCTCTCTCTCGCT | GGAGTAGAAATGACAGCGAGAGAGAGC |
| Vv-miR529 | TTTTTTTTTTAGCTGTACTCTCTCTCT | GGAGTAGAAAAGAAGAGAGAGAGTACA |
| Vv-miR827 | TTTTTTTTTTTGTTTGTTGATGATCAT | GGAGTAGAAATTAGATGATCATCAACA |
| Vv-miR828a | TTTTTTTTTTTGGAATACTCATTTGAG | GGAGTAGAAATCTTGCTCAAATGAGTA |
| Vv-miR828b | TTTTTTTTTTTGGAATACTCATTTGAG | GGAGTAGAAATCTTGCTCAAATGAGTA |
| Vv-miR845a | TTTTTTTTTTTATCAATTGGTATCAGA | GGAGTAGAAATAGCTCTGATACCAATT |
| Vv-miR845a* | TTTTTTTTTTCATCAATTGGTATCAGA | GGAGTAGAAATGCATGCTCTGATACCA |
| Vv-miR845b | TTTTTTTTTTTATCAATTGGTATCAGA | GGAGTAGAAATAGCTCTGATACCAATT |
| Vv-miR845b* | TTTTTTTTTTCATCAATTGGTATCAGA | GGAGTAGAAAGCATGCTCTGATACCAA |
| Vv-miR845c (845-3P) | TTTTTTTTTTCATCAATTGGTATCAGA | GGAGTAGAAAAGGCTCTGATACCAATT |
| Vv-miR1030a*, b* | TTTTTTTTTTCAGGTGCAGGTGCAAAT | GGAGTAGAAATCTGCATTTGCACCTGC |

Notes: MSP1 is the specific primer for miR-5’ RACE, and the underlined region is that pairs with the 3’ poly(A)n; MSP2 is the specific primer used for miR-3’ RACE, and the underlined region pairs with the 5’ adaptor.
