# Supplementary material for: Characterization of microRNAs Identified in a Table Grapevine Cultivar with Validation of Computationally Predicted Grapevine miRNAs by miR-RACE
Source: PLoS One. 2011 Jul 28;6(7):e21259. doi: 10.1371/journal.pone.0021259 (PMC3145640; doi:10.1371/journal.pone.0021259)
Supplement: Table S5 — Primers used for real-time PCR of Vv-miRNAs. (DOC) [file pone.0021259.s006.doc]

| **Table S5** | | |
| --- | --- | --- |
| **Vv-miRNAs** | **Primers for real-time PCR (5’→3’)** | **Length (nt)** |
| Vv-miR156b | UGACAGAAGAGAGUGAGCAC | 20 |
| Vv-miR159a | UUGGAGUGAAGGGAGCUCUC | 20 |
| Vv-miR169 | TGAGCCAAGGATGACTTGCCG | 21 |
| Vv-miR171j | TTGATTGAGCCGTGCCAATATC | 22 |
| Vv-miR172c | UUGAGCCGUGCCAAUAUCACG | 21 |
| Vv-miR393 | TCCAAAGGGATCGCATTGATC | 21 |
| Vv-miR394 | TTGGCATTCTGTCCACCTCC | 20 |
| Vv-miR398 | TGTGTTCTCAGGTCGCCCCTG | 21 |
| Vv-miR399 | TGCCAAAGGAGATTTGCTC | 19 |
| Vv-miR535i | TGACAGCGAGAGAGAGCACAC | 21 |
| Vv-miR827 | TTAGATGATCATCAACAAAC | 20 |
| R16328 | ATTCTAGAGGCCGAGGCGGCCGACATG | 27 |
