# Supplementary material for: Characterization of microRNAs Identified in a Table Grapevine Cultivar with Validation of Computationally Predicted Grapevine miRNAs by miR-RACE
Source: PLoS One. 2011 Jul 28;6(7):e21259. doi: 10.1371/journal.pone.0021259 (PMC3145640; doi:10.1371/journal.pone.0021259)
Supplement: Table S6 — Primers used for real-time PCR of target genes. (DOC) [file pone.0021259.s007.doc]

| **Table S6** | | |
| --- | --- | --- |
| **Target genes** | **Upstream primers (5’→3’)** | **Downstream primers (5’→3’)** |
| GR905745.1 | TGGTCCTGGAATTCCTTCAG | ATCCTGAAGGGGCATCCAT |
| CB920070.1 | TTGCTGGCATGCAGGGTA | TTCTTCACCGCCACCTTTAC |
| EC947732.1 | AGTTGGAAATGGGAAAGGCAGAG | TGGTGGCGGTGGGACTTATGG |
| FC065533.1 | AGCTGGTCGAGACGGGGAATA | GAAGAATGAGGCGAGGTGGC |
| CB917086.1 | AGCTGGTCGAGACGGGGAATA | GAAGAATGAGGCGAGGTGGC |
| EE102914.1 | GCCACAAACCATCAACAATG | AGACCCTCCTGACCCATACA |
| GR903243.1 | GATAGTGACAAGGGAATGCT | AAGGGTAACTTCACAGGATG |
| CF214477.1 | CATCAACTGGGTCAGGAGTG | GTGAGGACTGGGTCAACTAC |
| EX890146.1 | TATCCCTCTAGGCGAGCAAA | TATCCCTCTAGGCGAGCAAA |
| FC065533.1 | AGATGGGAATGTGAAATCTGG | TTCAGGCTTCTCAGCAAGCT |
| EC954557.1 | CACACCCATGATGAGAGAGG | CATATTCATAGCTGCAAAACACA |
